# A random effects model for the identification of differential splicing (REIDS) using exon and HTA arrays

**DOI:** 10.1186/s12859-017-1687-8

**Published:** 2017-05-25

**Authors:** Marijke Van Moerbeke, Adetayo Kasim, Willem Talloen, Joke Reumers, Hinrick W. H. Göhlmann, Ziv Shkedy

**Affiliations:** 1 Interuniversity Institute for Biostatistics and Statistical Bioinformatics, Hasselt University, Hasselt, 3500 Belgium; 2 Wolfson Research Institute for Health and Wellbeing, Durham University, Durham, UK; 3 Janssen Pharmaceutica, Beerse, 2340 Belgium

**Keywords:** Exon arrays, HTA arrays, Alternative splicing, Mixed effects models

## Abstract

**Background:**

Alternative gene splicing is a common phenomenon in which a single gene gives rise to multiple transcript isoforms. The process is strictly guided and involves a multitude of proteins and regulatory complexes. Unfortunately, aberrant splicing events do occur which have been linked to genetic disorders, such as several types of cancer and neurodegenerative diseases (Fan et al., Theor Biol Med Model 3:19, 2006). Therefore, understanding the mechanism of alternative splicing and identifying the difference in splicing events between diseased and healthy tissue is crucial in biomedical research with the potential of applications in personalized medicine as well as in drug development.

**Results:**

We propose a linear mixed model, Random Effects for the Identification of Differential Splicing (REIDS), for the identification of alternative splicing events. Based on a set of scores, an exon score and an array score, a decision regarding alternative splicing can be made. The model enables the ability to distinguish a differential expressed gene from a differential spliced exon. The proposed model was applied to three case studies concerning both exon and HTA arrays.

**Conclusion:**

The REIDS model provides a work flow for the identification of alternative splicing events relying on the established linear mixed model. The model can be applied to different types of arrays.

**Electronic supplementary material:**

The online version of this article (doi:10.1186/s12859-017-1687-8) contains supplementary material, which is available to authorized users.

## Background

Alternative splicing (AS) was considered to be an uncommon phenomenon until microarray and high-throughput sequencing technology enabled whole genome expression profiling [1]. More than 90% of human genes exhibit multiple transcript isoforms due to exon enrichment or depletion in mRNA transcription [2–4]. Since transcript isoforms of a single gene have been observed to vary between tissues and even between developmental stages, alternative splicing has been proposed as a primary driver of evolution and phenotypic complexity in mammals [5–7]. Straying splice variants, however, has been linked to cancers such as mammary tumorigenesis and ovarian cancer [8]. Although the underlying relationship between aberrant splicing events and cancer is often not (yet) established, the potential exists to develop new diagnostic and therapeutic interventions when more insights are gained [9]. Therefore, a better understanding of the mechanism of alternative splicing and identification of the differences in splicing events between diseased and healthy tissues is considered crucial in cancer and other medical research [10]. By measuring a relative amount of distinct splice forms, one can test whether a new splice form really constitutes an important fraction of a gene’s transcript in at least some cell types. This type of research could reveal patterns of regulation across a large number of different tissues [11]. Several alternative splicing detection methods have been proposed with the development of the RNA sequencing (RNASeq) [12] and microarray platforms such as the Affymetrix Exon ST arrays [13] and the Human Transcriptome Arrays 2.0 [14]. Recent studies emphasize the complementary nature of RNASeq and microarrays; combined, both technologies have strengths which might overcome the reported weaknesses. The primary advantage of RNASeq is its potential to explore the entire diversity of the transcriptome while the microarray has the ability to measure lower abundance transcripts [15]. Since the RNASeq is not able to properly account for low abundance transcripts and its competitive detection, the resulting library diversity will be limited [16, 17]. The limited diversity can be resolved by relying on the technology of exon and HTA arrays. Methods for alternative splicing detecting using RNASeq include Mats, DEXSeq and Cufflinks [18–20]. However, these have shown to be insufficient [21]. Alternative splicing has been studied with microarray platforms as well resulting in a variety of methods. The Microarray Detection of Alternative Splicing (MiDAS) method employs gene-level normalized exon intensities in an ANOVA model based on a Splicing Index (SI) [13, 22]. The SI method normalizes the exon level expression intensities by their corresponding gene level intensities, and compares these normalized intensities between sample groups. Another ANOVA based method is the so-called Analysis Of Splice VAriation (ANOSVA) [23], which fits a linear model to the observed data aiming to identify non-zero interaction terms between the sample groups and the exons. However, it has been argued that the ANOSVA method performed poorly [13]. The Probe Level Alternative Transcript Analysis (PLATA) method is based on the normalization of probe level intensities: first the probe-wise intensities, using gene level summarized values, are computed; afterwards the group averages of these normalized intensities are compared by considering all measurements across probes and arrays as independent [24]. The probe level SI estimation procedure for detecting differential splicing (PECA-SI method) detects alternative splicing based on a probe level splicing index instead of the exon level used by MiDAS [25]. PECA-SI outperforms other existing methods except for Finding Isoforms using Robust Multichip Arrays (FIRMA) [25, 26]. In contrast to other methods, FIRMA formulates alternative splicing identification as an outlier detection problem. It is based on the residuals of the Robust Multichip Analysis (RMA) [27]. A recent method is Robust Alternative Splicing Analysis for Human Transcriptome Arrays (RASA) [28] which was applied to HTA arrays and uses exon junction information in the identification of alternative splicing. In this paper, we propose a new modelling approach for the detection of AS namely the Random Effects for the Identification of Differential Splicing (REIDS). This model identifies splicing events based on a set of two scores; an *array score* which is used to identify samples containing an alternatively spliced exon and an *exon score* to prioritize spliced exons. The array scores have an intuitive interpretation as the deviation of the exon from the overall gene expression. The REIDS method was compared with FIRMA as the existing preferred method for alternative splicing detection using simulated data and two real-life exon array studies. A third case study based on HTA illustrates how the REIDS method enables the disentanglement of differentially expressed genes and differential spliced exons. The data and the proposed random effects model are introduced in the Methods sections. Next, the model is applied to three case studies in the Results sections. The paper is concluded with a discussion and conclusion. Illustrations are based on the R packages BiomaRt and GenomeGraphs [29]. REIDS is currently bundled in a package publicly available on R-forge.

## Methods

### Data

Three data sets are used to illustrate the proposed random effects model for the identification of alternative splicing.

#### The tissue data

The tissue data was obtained with the GeneChip®; Human Exon 1.0 ST array. The array is a whole genome array containing only perfect matching (PM) probes with a small number of generic mismatching probes for the purposes of background correction. A probe set identifies an exon using four perfect match probes. There are no probes which span exon-exon junctions [30]. The data set consists of triplicates from 11 tissues, so in total 33 arrays. Each tissue is thus represented by three replicates. This data set was also used to illustrate the FIRMA method [26] and is publicly available on the Affymetrix website.

#### The colon cancer data

The colon cancer data was also generated with the GeneChip®; Human Exon 1.0 ST array and contains 10 paired tumor-normal cancer samples. The data was analyzed before [9, 26] and is publicly available on the Affymetrix website.

#### The HTA data

The Human Transcriptome Array (HTA) is a recent microarray platform of Affymetrix. It is an expansion of the Human Exon array containing 10 probes per probe set. In addition, the HTA array contains probes that span exon-exon junctions which are supported by four probes each. The data was provided by Janssen Pharmaceutica, Belgium and contains measurements on seven tissues with three replicates each. An annotation file connecting the exon level to the gene level was taken from the Brainarray website [31]. As the provided cdf file currently does not yet annotate the junctions on the array, exon junctions are not considered in this paper.

### Models for the detection of alternative splicing

In this section we present the REIDS model for the detection of alternative splicing.

#### Finding Isoforms using Robust Multichip Arrays (FIRMA)

We begin with a brief description of the FIRMA model. The FIRMA algorithm for the detection of alternative splicing events relies on the RMA preprocessing approach [26, 27]. The algorithm consists of background correction, normalization and summarization of probe level data into gene level data, with one value per combination of gene and array. The gene level summarization is done by fitting an additive model on probe intensities:

1 $$ Y_{ij} = c_{i}+p_{j}+\epsilon_{ij}.  $$

Here, *Y*_*ij*_ denotes a log2-transformation of the intensities of array *i* and probe *j*. The parameter *p*_*j*_ denotes the average value of probe *j*, *c*_*i*_ represents the summarized gene level intensity of array *i* while the residual of probe *j* of array *i* is denoted by *ε*_*ij*_. The unknown parameters in the model are estimated using a median polish algorithm to ensure robust estimates of the summarized gene level intensities against outlying probes. The RMA model for summarization at the gene level can be extended to summarization at the exon level:

2 $$ Y_{ijk} = c_{i}+e_{k}+d_{ik}+p_{j}+\epsilon_{ijk}.  $$

The effect of exon *k* is denoted by *e*_*k*_ while *d*_*ik*_ represents the interaction between array *i* and exon *k* and *ε*_*ijk*_ is the residual of probe *j* which belongs to exon *k* in array *i*. Since the probes are nested within exons, the exon effect *e*_*k*_ is absorbed into the probe effect *p*_*j*_. Ignoring the interaction between the exon and the array, the information about alternative splicing is left to be absorbed into the residual [26]. This is a crucial point since it implies that alternatively spliced exons will have substantial higher residuals for some arrays than for others which motivates the definition of the FIRMA score as

3 $$ F_{ik}=median\,\,\, \epsilon_{ijk}/s.  $$

Here, probe *j* is assumed to belong to exon k (*j*=1,…*n*_*k*_) and *s* is the MAD (Median Absolute Deviation) allowing comparisons across genes. An exon is declared AS whenever *F*_*ik*_ is large [26].

#### The REIDS model

The alternative splicing detection problem can be formulated as a variance decomposition problem in a random effects model. The underlying assumption is that the between array variability of an alternatively spliced exon will be higher than the within array variability among the exons of the same gene. Similar to FIRMA, we define a linear model for the probe intensities:

4 $$  Y_{ijk} = p_{j}+d_{ik}+\epsilon_{ijk}.  $$

**Fig. 1:**
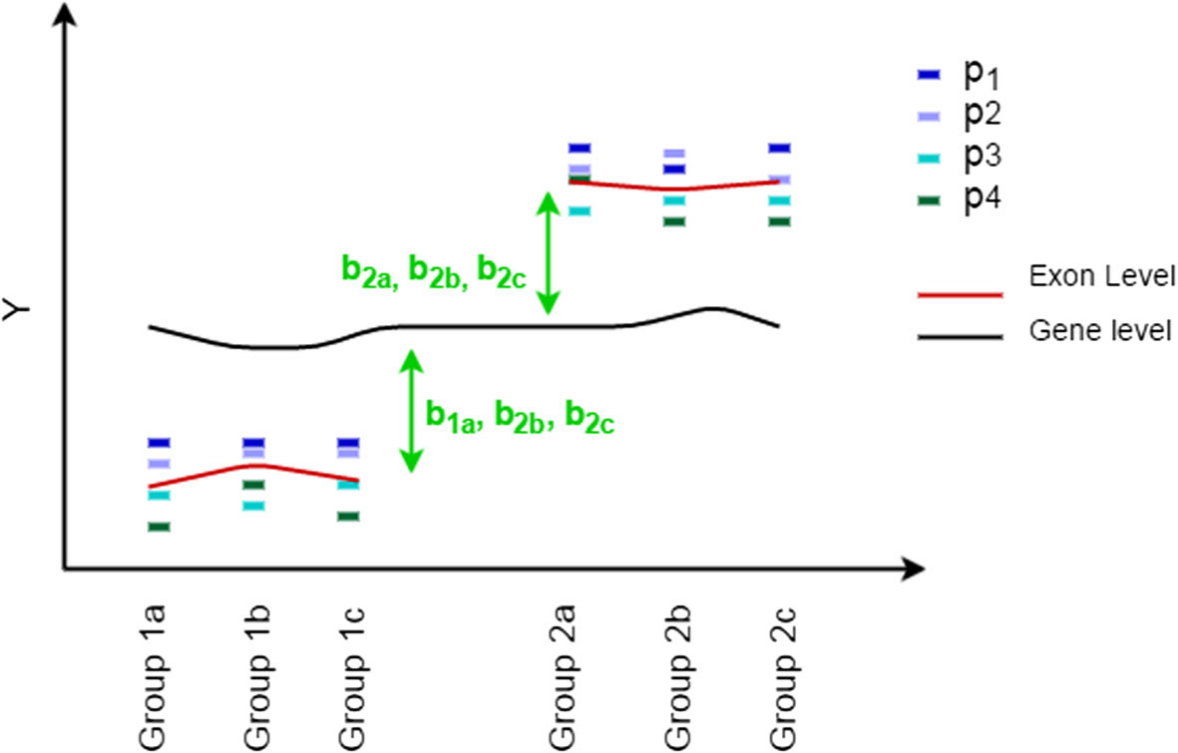
A clarification of the parameter estimation by the REIDS model

The background noise is assumed to follow a normal distribution, *ε*_*ijk*_∼*N*(0,*σ* ^2^) and it captures the within array variability (*σ* ^2^) across all exons of the same gene. In contrast to the FIRMA model, the parameter *d*_*ik*_ is decomposed into an average gene intensity per array *i*, *c*_*i*_, and an exon specific deviation from its average gene intensity *b*_*ik*_,

5 $$  d_{ik} = c_{i}+b_{ik}.  $$

where *b*_*ik*_∼*N*(**0**,**D**). The covariance matrix **D** is a *K*×*K* diagonal matrix containing the between array variabilities $\left (\tau ^{2}_{k}\right)$ for each exon. The model formulation in Eqs. (4) and (5) can be combined into a single model consisting of both the fixed effects (*p*_*j*_ and *c*_*i*_) and the random effects (*b*_*ik*_). The combined mixed effect model is given by:

6 $$ Y_{ijk} = p_{j}+c_{i}+b_{ik}+\epsilon_{ijk},  $$

in which the random effects *b*_*ik*_∼*N*(0,**D**) are assumed to be independent of the background noise *ε*_*ijk*_∼*N*(0,*σ* ^2^). Figure 1 illustrates the mean structure of the REIDS model presented in (5) for a scenario in which the gene is not differentially expressed and the *k*th exon is alternatively spliced. The exon is related to four probes. This results in four probe effects *p*_1_, *p*_2_, *p*_3_ and *p*_4_ which represent an average of the probe values across all arrays. The array effects in the REIDS model *c*_1*a*_, *c*_1*b*_, …, *c*_2*b*_ are used to measure the differences between the arrays. The deviation of the probes from the gene level will be captured by a random effect per sample: *b*_1*ak*_, *b*_1*bk*_, …, *b*_2*ck*_ which are, as mentioned above, assumed to follow a normal distribution with variability *τ*_*k*_. The remaining variation of a probe *j* of exon *k* in array *i* is captured by the error term *ε*_*ijk*_. Hence, the model splits the total variability of the probe intensities of an exon *k* into the variability which can be accounted for by the arrays $\tau ^{2}_{k}$ and an the remaining variability *σ* ^2^.

**REIDS Scores for Quantification of Alternative Splicing** The advantage of a mixed model formulation for alternative splicing detection is the existence of a standard score for every exon in every sample which quantifies the trade-off between signal and noise. We refer to this score as the *exon score*. The exon score for the *k*th exon in a gene is defined as:

$$\rho_{k}= \tau^{2}_{k}/\left(\sigma^{2}+\tau^{2}_{k}\right). $$

It intuitively follows from this definition that an equity threshold for the exon score is 0.5. Note that this threshold can be adapted depending on the amount of signal in a microarray data set. Given that exon *k* has been identified to have substantial variation between the arrays, the estimated random effects *b*_*ik*_ per array per exon can be used as array scores to quantify the degree of alternatively splicing per array. Arrays enriched or depleted with exon *k* will have array scores greater than zero. It should be noted that the array scores are expected to be correlated with the FIRMA scores for an alternatively spliced exon as both the random effects of the REIDS model will resist and the residuals of the FIRMA model will be large. The combination of an exon score and an array score gives enables us to differentiate between differential expression of a gene and differential splicing of an exon. Four scenarios can be distinguished for which illustrations can be found in Section 2 of Additional file 1.

- The *first scenario* describes a gene that is not differentially expressed between the arrays and has no alternatively spliced exons. This implies that exon intensities are similar across all arrays. In this case it is expected that $\tau ^{2}_{1}=\ldots =\tau ^{2}_{K}=\tau ^{2}$ and *τ* ^2^<<*σ* ^2^. As a consequence, the exon score *ρ*_*k*_ will be low and the exons should not be identified by the model.
- The *second scenario* consists of a non-differentially expressed gene that contains an alternatively spliced exon *k* and non-alternatively spliced exons *k*−. For the alternatively spliced exon, it is expected that $\tau ^{2}_{k} > \tau ^{2}_{k-}$ with $\tau ^{2}_{k} >> \sigma ^{2}$ and $\tau ^{2}_{k-} << \sigma ^{2}$. The exon score for this probe set *k* will be high. As an acceptable *ρ*_*k*_ is present, a test on the array scores can be conducted in order to identify biologically induced splicing associated with the experimental conditions or tissue types.
- The *third scenario* corresponds to a differentially expressed gene with no alternatively spliced exons. Again it is expected that $\tau ^{2}_{1}=\ldots =\tau ^{2}_{K}=\tau ^{2}$. Since there is a natural difference between the gene levels of the arrays here; it will be the case that *τ* ^2^>>*σ* ^2^ and that the exon scores are high. A test on the array scores will conclude the absence of alternatively spliced exons since the scores will not be associated with experimental conditions or tissue types.
- The *fourth scenario* is a differentially expressed gene with an alternatively spliced exon. For the alternatively spliced exons, the same reasoning applies as for when the gene is not differentially expressed. The non-alternatively spliced exons will show enough signal in the exon score but a test between the array scores will show no association with experiment conditions or tissue types.

**Estimation of the Model Parameters** The parameters of the proposed mixed effects model are estimated within the Bayesian framework with vague proper priors since the full conditional posterior distributions for the parameters of interest are known. Let **D** be a *K*×*K* diagonal covariance matrix of $\tau ^{2}_{1},\tau ^{2}_{2},\cdots,\tau ^{2}_{K}$ for which an Inverse-Wishart prior was assumed, i.e., **D**∼*Inverse*−*Wishart*(*ψ*,**Ω**). An inverse gamma prior was specified for *σ* ^2^ and 1/*σ* ^2^∼*Gamma*(*α*,*β*). The full conditional posterior distributions for the parameters of interest are given by

$$P\left(\mathbf{b_{i}}|\mathbf{p},\mathbf{c},\mathbf{D},\sigma^{2}\right) = N_{K}\left(\mathbf{\Phi},\mathbf{\Upsilon}^{-1}\right). $$

Here, **Υ**=*D* ^−1^+*σ* ^−2^ *n*_*i*_ where *n*_*i*_ is a *K* vector of number of probes per exon. Further, **Φ**=**Υ** ^−1^ *Θ* ^′^ where **Θ** is a *K* vector of $\sigma ^{-2}\sum _{j,k} (log2(PM_{ijk(j)})-p_{k}-c_{i})$. Hence, the full conditional posterior distribution for **D**, the matrix of the between array variability is

$$P\left(\mathbf{D}|\mathbf{b},\mathbf{p},\mathbf{c},\sigma^{2}\right) = Inverse-Wishart(\psi + n,\mathbf{\Omega}+\mathbf{b}'\mathbf{b}), $$

where **Ω** is a *K*×*K* diagonal matrix of ones, *n* is the number of arrays with *ψ* specified as the number of exons. Finally, the full conditional distribution for 1/*σ* ^2^ is

$$P(1/\sigma^{2}|\mathbf{b},\mathbf{p},\mathbf{c},\mathbf{D}) = Gamma(\alpha+0.5N,\eta) $$

where $\eta = \beta +0.5\sum _{i,j,k}(Y_{ijk(j)}-p_{k}-c_{i}-b_{ik}z_{ik})^{2}$ with *α*=*β*=0.0001 and *N* is the number of observations for all the arrays, exons and probes. Using Gibb’s sampler, we generate posterior samples for the parameters by iteratively sampling from their full conditional posterior distributions conditioning on the sample of the parameters at the immediate previous iteration. The posterior point estimates and the credible intervals for the parameters are based on the MCMC chains after discarding the burn-in parts.

**Fig. 2:**
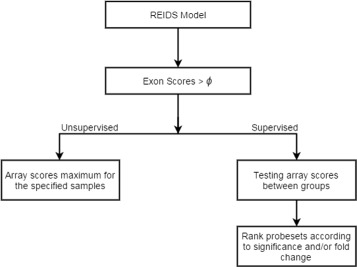
The REIDS method flowchart. The proposed workflow is similar to the workflow of FIRMA. Both models fit a statistical model on the PM data and compute a score on which the decision whether or not an exon is alternative splicing is based. In case of an AS event, we expect to see a correlation between the array scores of the REIDS model and the FIRMA scores

**Identification of Alternative Splicing Events** There are two main types of alternative splicing detections: (1) detection of sample-specific alternative splicing and (2) detection of differential splicing between two or more experimental conditions. Figure 2 illustrates the flexible framework of the mixed model and how it can be used to investigate either sample-specific alternative splicing or differential splicing between experimental groups. First the REIDS method is applied to each gene to obtain array and exon scores after which the probe sets are prioritized according to their exon scores. Probe sets with exon scores greater than a pre-specified threshold (0<*ρ*<1) are retained for further investigation. The exon scores directly reflect the heterogeneity between samples and consequently, a probe set with a high exon score implies enrichment or depletion of the exon in some of the samples. A prioritized probe set is considered to be expressed in a subset if the array scores for some samples are further away from zero compared to the other samples or if the samples have the maximum array score for exon enrichment or the minimum array score for exon depletion. For the detection of differential splicing between two or more experimental conditions, the exon scores also reflect heterogeneity between arrays. This does not imply that such heterogeneity is associated with experimental conditions. Heterogeneity between arrays captured by exon scores is a necessary but not a sufficient criterion for differential splicing detection. We recommend to use the array scores as input into a t-test for independent arrays or a paired t-test for paired arrays to test whether the array scores are significantly different between experimental conditions. Other relevant tests might also be performed as the framework is flexible and allows many types of downstream analyses. Finally, the prioritized exons are ranked according to their corresponding *p*-values or t-statistics.

**Exclusion of Non-Informative Probe Sets** Alternative splicing detection is known to suffer from a large number of false positives when many probes in a probe set are non-informative. Therefore, filtering has been recommended as a step prior to alternative splicing detection [9, 26]. A non-informative probe set can be defined by a lack of coherence among its probes. By evaluating the intra-probe set correlation, a non-responsive probe set can be identified as such and excluded prior to alternative splicing detection based on informative calls. The concept of informative or non-informative calls was introduced for arrays by applying a factor analysis model to calculate a score of informativeness based on signal to noise ratio [32]. We used a mixed model framework for Informative/Non-Informative calls (I/NI calls) to identify and exclude non-responsive probe sets based on an intra-probe set correlation as a filtering score [33].

## Results

In this section we present the analysis of the three case studies presented in “Background” section. All data sets are pre-processed using the R package aroma.affymetrix [34]. The raw.CEL files are background corrected with the RMA background correction, normalized with quantile-normalization and log2-transformed [27] resulting in probe level intensities on which first the I/NI calls and then REIDS model are performed. For the first case study, the tissue data, we illustrate the method on three genes for which several probe sets were identified to be alternative spliced. For the second case study, the colon cancer data, we present the results for 24 validated genes. The third case study, the HTA data, shows examples of the four scenarios described above.

### The tissue data

#### The ABLIM1 gene

**Fig. 3:**
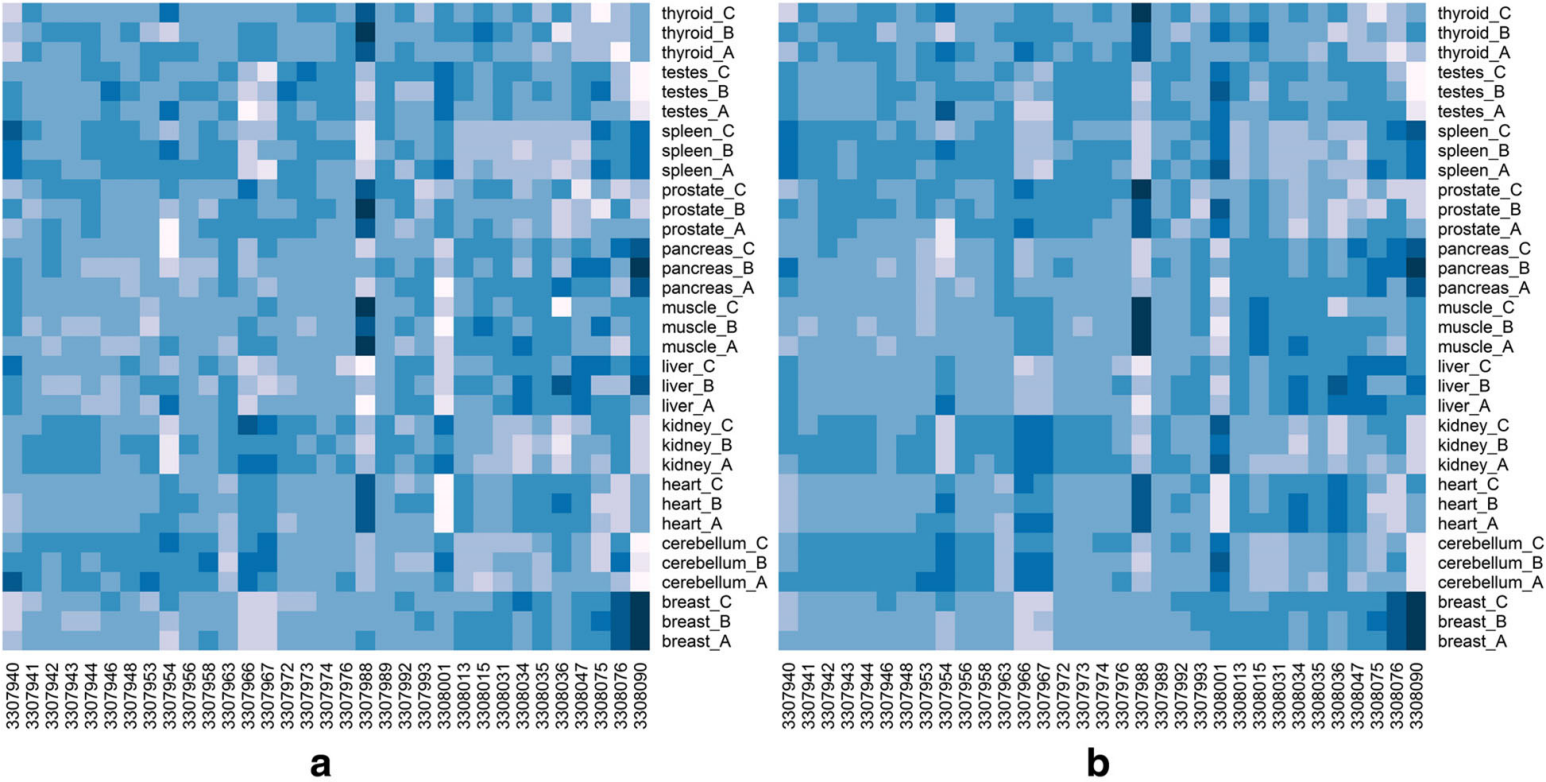
*Left panel*: a heatmap of the FIRMA scores of the ABLIM1 gene. *Right panel*: a heatmap of the array scores of the ABLIM1 gene

The tissue data contains 284258 probe sets for 18708 unique genes. In order to illustrate the methods, we first focus on the ABLIM1 gene which was validated to be alternatively spliced [9]. The ABLIM1 gene contains 35 probe sets, 33 of which pass the I/NI calls threshold. Figure 3 shows the FIRMA scores (log scaled) and array scores from the REIDS method. As expected, the array scores and the FIRMA scores are strongly correlated. Ten probe sets have exon scores greater than the equity threshold of 0.5 but only four have exon scores higher than 0.7. Probe set 3307988, which was validated in earlier studies and was also discovered by FIRMA, has the highest exon score of 0.82 with array scores ranging from -2 to 2.5 [9, 26]. REIDS also identified exon 3307988 as alternatively spliced for the heart and muscle tissues. The measured intensities of all probe sets of the ABLIM1 gene and the annotation to known transcripts can be found in Additional file 1: Figure S6.

#### A genome wide analysis

**Fig. 4:**
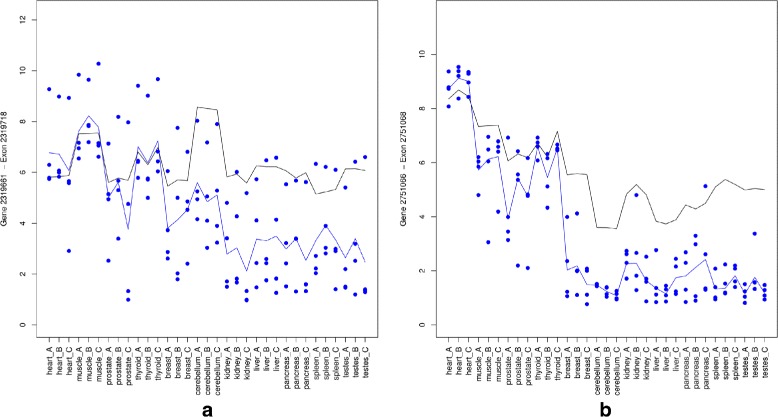
*Left panel*: the probe set 2319718 of the KIF1B gene. *Right panel*: the probe set 2751068 of the PALLD gene. The *black* and *blue lines* indicate the mean profiles of the gene and exon level data respectively. The *blue dots* show the probe level data

A genome wide analysis was conducted on the tissue data considering the heart, muscle, prostate and thyroid tissues as one group and the remaining tissues as another group. In total 1334 of the 4579 probe sets with exon scores exceeding 0.5 are identified to be alternatively spliced between tissues using the t-test with a BH-FDR false discovery correction [35] using an error rate of 5%. In what follows we focus on two examples. The top ranked probe set (based on the adjusted *p*-values) is 2513813 with an exon score of 0.76, which maps to the XIRP2 gene. This probe set and annotation of the gene to known transcripts are shown in Additional file 1: Figure S7 and S8. Probe set 2319718 from gene KIF1B is found to be up-regulated in the heart, muscle, thyroid and prostate tissues as well, but depleted in the other tissues. The KIF1B gene has previously been reported to be differentially expressed in 32 cancer experiments and to be alternatively spliced in heart, muscle and thyroid [36]. A third example is the PALLD gene which has been found in 75 cancer experiments and whose probe sets (2751068 and 2751072) were identified by REIDS to be up-regulated in heart, muscle, thyroid and prostate, but down-regulated in the other tissues. Figure 4 shows the gene level and exon level data for probe set 2319718 from the KIF1B gene and 2751068 from the PALLD gene.

### The colon cancer data

**Fig. 5:**
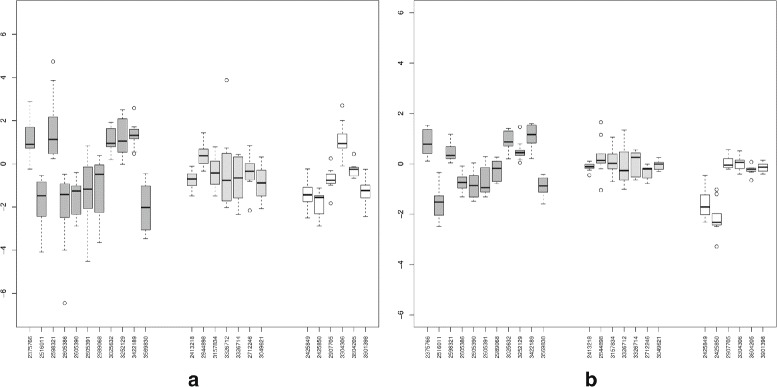
*Left panel*: the mean paired differences of the FIRMA scores for the 24 validated probe sets. *Right panel*: the mean paired differences of the array scores for the 24 validated probe sets. *Dark grey boxes* indicate confirmed probesets while *light grey boxes* are unconfirmed probesets and *white boxes* are unclear probesets

**Fig. 6:**
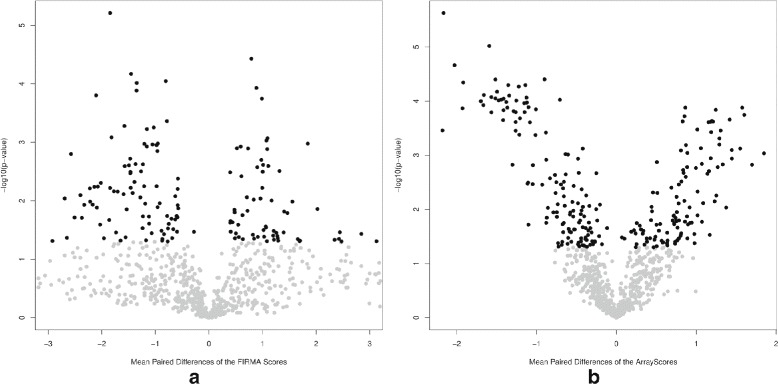
*Left panel*: a vulcano plot of the -log10(*p*-values) versus the mean paired differences of the FIRMA scores. *Right panel*: vulcano plot of the -log10(*p*-values) versus the mean paired differences of the array scores for the probe sets with an exon score higher than 0.5

The colon cancer data contains 10 paired tumor-normal cancer samples and 284258 probe sets from 18708 uniquely identified genes. The goal of the analysis is to identify exons whose differential splicing can be associated with tumors or normal samples. The paired t-test was used to test whether the mean paired differences of the array scores is equal to zero or not. First, we focus on the 24 validated probe sets [9] of which 11 probe sets were ‘confirmed’ to be alternatively spliced, seven probe sets were ‘unconfirmed’ and six were categorized as ‘unclear’. With the term ’confirmed’ we refer to probe sets which have been confirmed to be alternatively spliced by RT-PCR results to consistently have a different isoform in cancer from the normal [26]. Figure 5 presents the fold change from the FIRMA and REIDS methods. The FIRMA scores and the array scores obtained by the REIDS method for these probe sets are strongly correlated. The FIRMA scores are observed to contain more noise. The array scores for the ‘confirmed’ probe sets are more dissociated from zero as compared to the ‘unconfirmed’ and the ‘unclear’ probe sets. A genome-wide scan for differential splicing on the colon data identified 894 probe sets with exon scores greater than 0.5. Figure 6 shows the volcano plots of the *p*-values and fold changes for the FIRMA and REIDS methods. The most interesting probe sets with evidence of tumor induced differential splicing are located in the upper left and right corners of the plots. These are the probe sets with the largest fold changes and the smallest *p*-values. A total 114 of probe sets were identified as alternatively spliced (using a significance level of 5%). A further comparison can be found in Section 4 of Additional file 1.

### The HTA data

The HTA data contains 36799 genes and 575650 probe sets. The cancer cell lines were grouped into two groups. The first group contains the colon cancer cell lines (HCT-116 and HT-29) and the second group the cell lines from lung (A549 and NCI-H460), ovary (SK-OV-03), prostate (DU-145) and breast cancer (MDA-MB-231) cell lines. This division is clear using a spectral map analysis shown in Section 5 of Additional file 1. A genome-wide analysis of the HTA data resulted in 2522 probe sets that are likely to be alternatively spliced between the colon tissues and the other tissues (ovary, prostate and breast). The top ranked probe set is ENSE00001668645 with an exon score of 0.70 which is presented in the Additional file 1: Figure S17. This exon is mapped to the DOCK10 gene which has been reported in several cancer studies [37].

#### A differentially expressed gene with an alternatively spliced exon

**Fig. 7:**
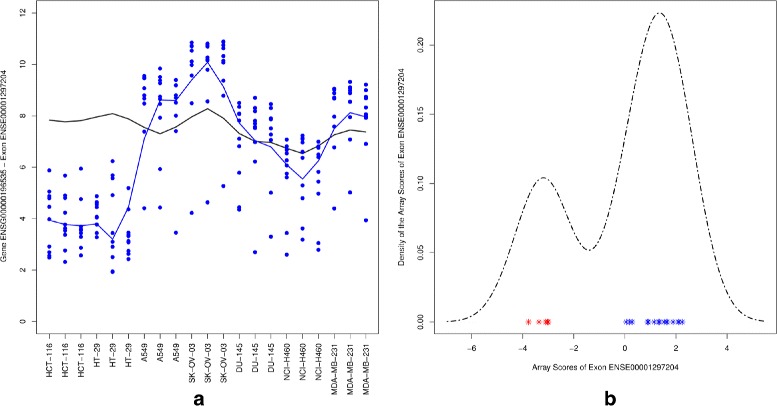
Probe set ENSE00001297204. *Left panel*: gene level and exon level data. The *black* and *blue lines* indicate the mean profiles of the gene and exon level data respectively. The *blue dots* show the probe level data. *Right panel*: a density plot for array scores showing the values of group 1 (*red*) and group 2 (*blue*)

Figure 7 (left panel) shows the gene level data of the MYO18A gene with the exon level data of probe set ENSE00001297204. The gene was significantly differentially expressed between colon and other cell lines with a p-value of 0.0008 and fold change of 0.57. The fold change for the gene level data was however much smaller than the fold change at the exon level. This indicates that this particular exon behaves differently compared to the other exons of the same gene. The ability to separate signal (i.e. variability between samples) from noise, is one of the main advantage of the REIDS method over FIRMA. The density plot of the array scores of ENSE00001297204 (Fig. 7 b) shows the clear separation between the colon cell lines and the other cell lines. This superimposed bimodal distribution of the array scores illustrates the discrimination of the random effects model for alternating splicing detection.

#### A differentially expressed gene with non alternatively spliced exon

**Fig. 8:**
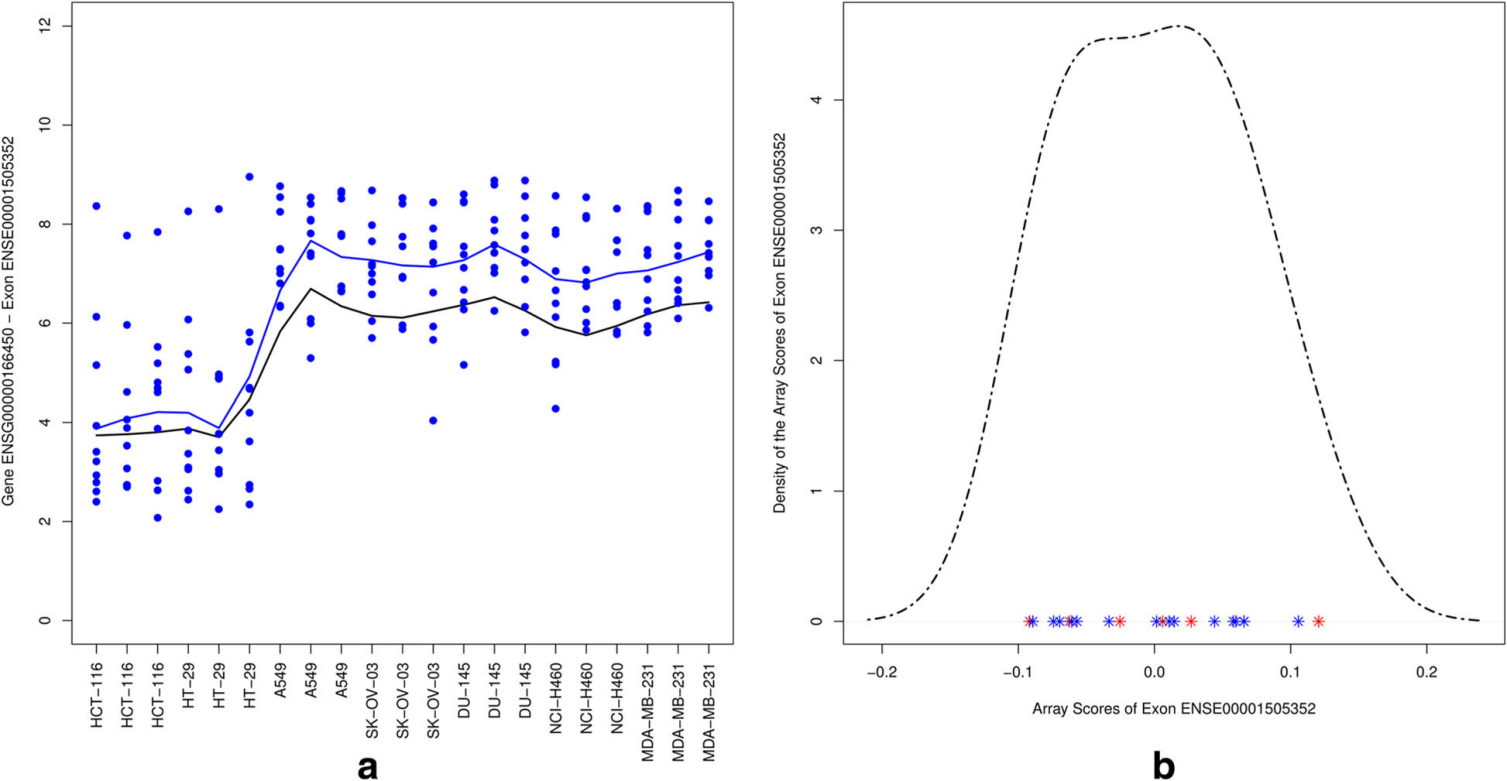
Probe set ENSE00001505352. *Left panel*: gene level and exon level data. The *black* and *blue lines* indicate the mean profiles of the gene and exon level data respectively. The blue dots show the probe level data. *Right panel*: a density plot for array scores showing the values of group 1 (*red*) and group 2 (*blue*)

In the previous example we focused on an alternative spliced exon for a differentially expressed gene. This section shows an example of a differentially expressed gene with no splicing variants. Figure 8 (left panel) shows the gene and exon level data for probe set ENSE00001505352 of the PRTG gene. Both gene level and exon level data show a similar pattern across the cell lines with fold changes of 2.40 and 3.11, respectively. This implies that this exon is expressed similarly as the others exons of the PRTG gene. We note that both gene level and the exon level data are lower for the colon cancer cell lines compared to the level in the ovary, prostate and breast cancer group. This implies that the gene is differentially expressed. Furthermore, Fig. 8 (right panel) shows a unimodal distribution for the arrays scores which implies that these are not discriminatory between colon and other tissues (i.e., the exon ENSE00001505352 is not alternatively spliced). Thus, the REIDS model is able to differentiate between differential gene expression and differential splicing.

#### A non differentially expressed gene with an alternatively spliced exon

**Fig. 9:**
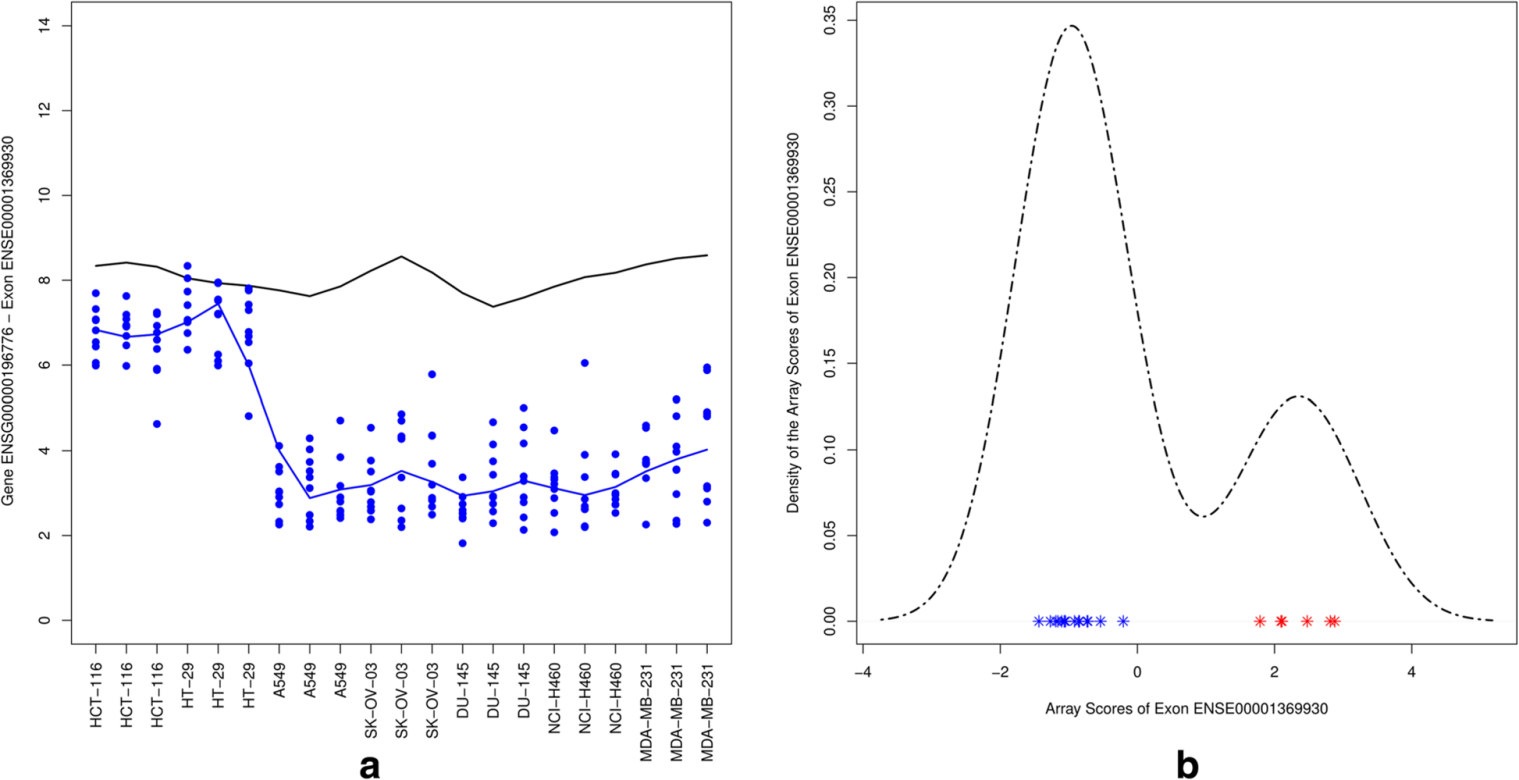
Probe set ENSE00001369930. *Left panel*: gene level and exon level data. The *black* and *blue lines* indicate the mean profiles of the gene and exon level data respectively. The *blue dots* show the probe level data. *Right panel*: a density plot for array scores showing the values of group 1 (*red*) and group 2 (*blue*)

In the next two sections we present examples for non-differentially expressed genes. Figure 9 shows an example of the non-differentially expressed gene CD47 of which probe set ENSE00001369930 with an exon score of 0.93 is alternatively spliced. The values of probe set ENSE00001369930 has consistently high expression in all colon cancer samples while it is expressed 6 fold lower in the other samples. The density plots of the array scores indicates consequently a clear bimodal distribution which represent a distinction between the groups of interest.

#### A not differentially expressed gene with a non alternatively spliced exon

**Fig. 10:**
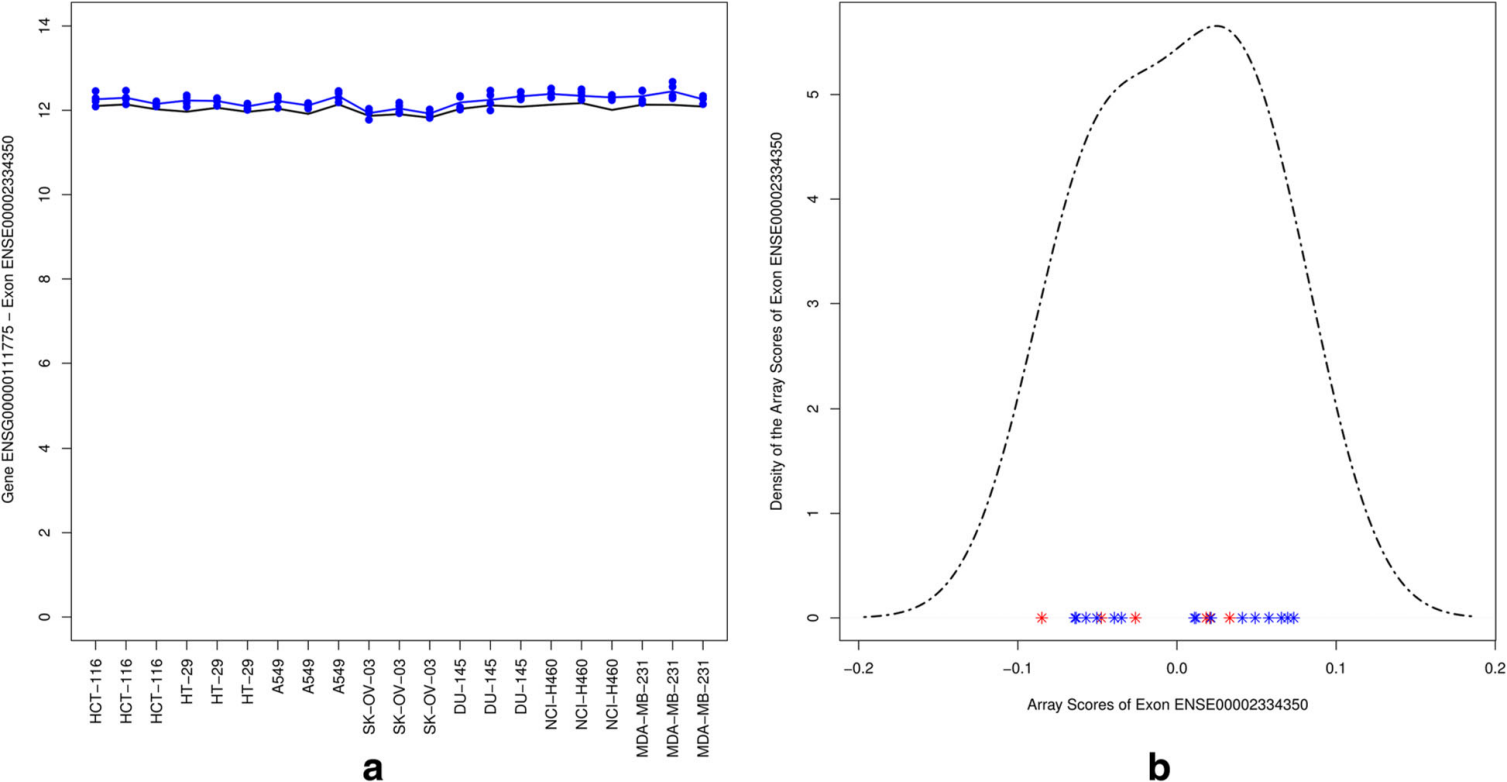
Probe set ENSE00002334350. *Left panel*: gene level and exon level data. The *black* and *blue lines* indicate the mean profiles of the gene and exon level data respectively. The *blue dots* show the probe level data. *Right panel*: a density plot for array scores showing the values of group 1 (*red*) and group 2 (*blue*)

As an illustration that REIDS successfully identifies genes without signal as negative outcomes, Fig. 10 shows a non-differentially expressed gene with a non-alternatively spliced exon. The array scores of probe set ENSE00002334350 with an exon score of 0.79 are not significantly different between the groups of interest. The probe set belongs to the COX6A1 gene. The density plot of the array scores resembles a unimodal distribution and does not show a distinction between the groups of interest.

### Simulation study

Although a mixed effect model is a well-established statistical methodology, a simulation study using the same setting as Purdom et al. (2008) should be completed, in order to test its usage for alternative splicing detection. The data are simulated from the following model:

7 $$ y_{ij} = log2\left(B_{j} + I_{ij}\times 2^{(Ci+Pj)} + \epsilon_{ij}\right)   $$

where *y*_*ij*_ denotes the intensity for array *i* and probe *j*. **B**_*i*_∼*N*(5,0.35^2^) is the background noise common to all arrays and all probes. *P*_*j*_∼*N*(0,3) denotes probe specific effects whilst *ε*_*ij*_∼*N*(0,0.7^2^) denotes the residuals from array *i* and probe *j*. The array mean effect *c*_*i*_∼*N*(*c*,1.5^2^) was assumed to have two mean values *μ*_*c*_={7,10} with a common standard deviation of 1.5. Each of the simulated alternatively spliced genes contained 40 arrays and 10 exons with four probes per exon. The spliced isoforms were randomly selected from a set of pre-defined patterns with equal probability and the arrays that contained a spliced isoform were randomly selected with probabilities (P = 0.1,0.3,0.5,0.8) [26]. In total 1000 datasets were generated. The results of the simulation study are presented in Table 1 for a probability of 80% for including a splice isoform. The REIDS method and the FIRMA method are comparable when there was a low probability of splice variants. However, the REIDS method outperformed the FIRMA method when there was a high probability of splice variants.

**Table 1:** Area under the curve for 80% probability of alternative splicing

Method	*μ*=7	*μ*=10
REIDS	0.97	0.99
FIRMA(mean)	0.90	0.94
FIRMA(median)	0.89	0.93

A second simulation study based on eqn 7 was performed in order to investigate the performance of informative calls in identification of non-responsive probe sets [38]. All arrays were simulated from the background noise with no array and probe effects, except for the arrays that were randomly selected with probabilities *P*=(0.05,0.1,0.15,0.2) to contain one or more non-responsive probe sets. Table 2 shows that informative calls as a filtering approach prior to alternative splicing detection correctly identified non-responsive probe sets in more than 90% of times independent of the number of non-responsive probe sets. By applying informative calls before alternative splicing detections, the problem of non-responsive probe sets could be minimized and consequently, a reduction in false positive rates.

**Table 2:** Area under the curve for non-responsive probe set identification

Non-responsive	Arrays			
probe sets	5*%*	10*%*	15*%*	20*%*
1	0.987	0.993	0.994	0.994
2	0.985	0.992	0.994	0.994
3	0.993	0.997	0.997	0.997
4	0.988	0.993	0.995	0.995
5	0.992	0.996	0.997	0.997
6	0.992	0.996	0.997	0.997

## Discussion

We have reformulated the identification of alternative splicing events in terms of a random effects model. Alternative splicing is seen as the deviation of the exon level data from its gene level data in a subset of samples or cell lines or under a set of conditions. The proposed REIDS method is capable of identifying cassette alternative exon usage which is the most prevalent type of alternative splicing [39, 40]. The identification relies on a set of scores: the exon score and the array scores produced by the model. Exons which are alternative splicing candidates will have larger exon scores implying that an alternatively spliced exon must be discriminatory between tissues or experimental conditions. In addition to exon scores, large positive array scores indicate exon enrichment while large negative values indicate exon depletion. Overall, REIDS is at least as good as FIRMA since both rely on a similar concept. REIDS, however, detects alternative splicing based on a signal-to-noise ratio instead of relying on the total variability as used by the FIRMA method. This means that both the REIDS and FIRMA methods will perform equally well when an exon is alternatively spliced. The main difference is that FIRMA is more prone to false positives than REIDS as shown in our simulation study. We have presented a number of alternatively spliced probe sets across the case studies which are supported by literature. For example, probe set 3307988 of the ABLIM1 gene in the tissue data was found by the REIDS model and confirmed by [9]. The aforementioned KF1B gene has been cited to be subjected to alternative splicing in heart, muscle and thyroid [2, 36] as well. In the colon cancer study, 11 confirmed alternatively spliced probe sets were identified before [9]. The REIDS model also identified six of these as alternatively spliced implying that we have not found all of the confirmed probe sets. Our results so far seem to coincide with literature for several probe sets.

## Conclusions

Alternative splicing detection is becoming an interesting area of methodological research in genomics with the introduction of exon arrays and RNA-sequencing platforms. A better understanding of variation in gene expression between tissue types or experimental conditions is a crucial element to advance precision medicine. The biological dogma of one gene leading to multiple proteins makes the investigation of alternative splicing detections appealing for drug development and target identification. Pharmaceutical research seeks new putative targets in cancer therapy with advances in biotechnologies and increasing knowledge of the genome. Our future work consists of improving and investigating the effects of the model. A next step could be to expand the model to a new summarization technique. In this way, we would have a model that is able to identify alternative splicing and summarize intensities at the gene and exon level. Concerning the HTA data, we will investigate if we can make use of the junction probes that are present on this array. In the above analysis, the HTA data was annotated with a cdf file, publicly available on the Brainarray website, and the junction probes were not detected in this file. We will endeavor to discover the junction probes and see in which way we can use them to our benefit. Once these can be annotated, we can compare our method to RASA which makes use of the junctions in the detection of alternative splicing. Finally, as an extension of the model, it will be interesting if we can perform a similar analysis for RNASeq data. Our goal is to see if this type of model can be applied to both microarray and RNASeq data with minor alterations. Studying the transcriptome might be most efficient, combining both technologies as suggested by the Sequencing Quality Control Consortium [41]. During the further development of the model and its applications, we will also develop an R package with our method and its elaborations to perform a data analysis in a pipeline.

## Additional file

Additional file 1 Supplementary Material. Supplemental examples and figures (PDF 31000 kb)

## Abbreviations

AS: Alternative splicing
SI: Splice index
ANOSVA: Analysis of splice variation
PLATA: Probe level alternative transcript analysis
RASA: Robust alternative splicing analysis
iGEMS: integrated gene and exon model of splicing
MiDAS: Microarray detection of alternative splicing
REIDS: Random effects for the identification of differential splicing
RNASeq: RNA sequencing
HTA: Human transcriptome array
FIRMA: Finding isoforms using robust multichip arrays
PM: Perfect matching
MAD: Median absolute deviation
I/NI: Informative /non-informative
RMA: Robust microarray analysis

## References

[1] Mironov AA, Fickett JW, Gelfand MS (1999). Frequent alternative splicing of human genes. Genome Res.

[2] Wang ET, Sandberg R, Luo S, Khrebtukova I, Zhang L, Mayr C, Kingsmore SF, Schroth GP, Burge CB (2008). Alternative isoform regulation in human tissue transcriptomes. Nature.

[3] Pan Q, Shai O, Lee LJ, Frey BJ, Blencowe BJ (2008). Deep surveying of alternative splicing complexity in the human transcriptome by high-throughput sequencing. Nat Genet.

[4] Chen L, Schölkopf B, Zhao H, Lu HH-S (2011). Statistical and Computational Studies on Alternative Splicing. Handbook of Statistical Bioinformatics.

[5] Black DL (2003). Mechanisms of alternative pre-messenger rna splicing. Ann Rev Biochem.

[6] Epstein CJ (1986). Developmental genetics. Experientia.

[7] Crayton ME, Powell BC, Vision TJ, Giddings MC (2006). Tracking the evolution of alternatively spliced exons within the dscam family. BMC Evol Biol.

[8] Fan W, Khalid N, Hallahan AR, Olson JM, Zhao LP (2006). A statistical method for prediciting splice variants between two groups of samples using genechip expression array data. Theor Biol Med Model.

[9] Gardina P, Clark T, Shimada B, Staples M, Yang Q, Veitch J, Schweitzer A, Awad T, Sugnet C, Dee S, Davies C, Williams A, Turpaz Y (2006). Alternative splicing and differential gene expression in colon cancer detected by a whole genome exon array. BMC Genomics.

[10] Bisognin A, Pizzini S, Perilli L, Esposito G, Mocellin S, Nitti D, Zanovello S, Bortoluzzi P, Mandruzzato S (2014). An integrative framework identifies alternative splicing events in colorectal cancer development. Mol Oncol.

[11] Lee C, Roy M (2004). Analysis of alternative splicing with micro-arrays: successes and challenges. Genome Biol.

[12] Wang C, Gong B, Bushel PR, Thierry-Mieg J, Thierry-Mieg D, Xu J, Fang H, Hong H, Shen J, Su Z, Meehan J, Li X, Yang L, Li H, Labaj PP, Kreil DP, Megherbi D, Gaj S, Caiment F, van Delft J, Kleinjans J, Scherer A, Devanarayan V, Wang J, Yang Y, Qian HR, Lancashire LJ, Bessarabova M, Nikolsky Y, Furlanello C, Chierici M, Albanese D, Jurman G, Riccadonna S, Filosi M, Visintainer R, Zhang KK, Li J, Hsieh JH, Svoboda DL, Fuscoe JC, Deng Y, Shi L, Paules RS, Auerbach SS, Tong W (2014). The concordance between rna-seq and microarray data depends on chemical treatment and transcript abundance. Nat Biotechnol.

[13] Affymetrix. Alternative transcript analysis methods for exon arrays: Affymetrix Whitepaper; 2005.

[14] Affymetrix. Genechip human transcriptome array 2.0 data sheet. 2013. Available at http://tools.thermofisher.com/content/sfs/brochures/hta_array_2_0_datasheet.pdf#/legacy=affymetrix.com. Accessed 19 Feb 2015.

[15] Sood S, Szkop KJ, Nakhuda A, Gallagher IJ, Murie C, Brogan RJ, Kaprio J, Kainulainen H, Atherton PJ, Kujala UM, Gustafsson T, Larsson O, Timmons JA (2016). igems: an integrated model for identification of alternative exon usage event. Nucleic Acids Res.

[16] Lei R, Ye K, Gu Z, Sun XA (2015). Diminishing returns in next-generation sequencing (ngs) transcriptome data. Gene.

[17] Mele M, Ferreira PG, Reverter F, DeLuca DS, Monlong J, Sammeth M, Young TR, Goldmann JM, Pervouchine DD, Sullivan TJ, Segre AV, Djebali S, Niarchou A, Wright FA, Calvo M, Getz G, Dermitzakis ET, Ardlie KG, Guigo R (2015). The human transcriptome across tissues and individuals. Science.

[18] Shen S, Park JW, Huang J, Dittmar KA, Zhou Q, Carstens RP, Xing Y, Lu Z-X (2012). Mats: a bayesian framework for flexible detection of differential alternative splicing from rna-seq data. Nucleic Acids Res.

[19] Anders S, Reyes A, Huber WA (2012). Detecting differential usage of exons from rna-seq data. Genome Res.

[20] Trapnell C, Williams BA, Pertea G, Mortazavi A, Kwan G, Salzberg SL, Wold BJ, Pachter L, van Baren MJ (2010). Transcript assembly and quantification by rna-seq reveals unannotated transcripts and isoform switching during cell differentiation. Nat Biotechnol.

[21] Liu R, Loraine AE, Dickerson JA (2014). Comparisons of computational methods for differential alternative splicing detection using rna-seq in plant systems. BMC Bioinformatics.

[22] Clark T, Schweitzer A, Chen T, Staples M, Lu G, Wang H, Williams A, Blume J (2007). Discovery of tissue-specific exons using comprehensive human exon microarrays. Genome Biol.

[23] Cline MS, Blume J, Cawley S, Clark T, Hu JS, Lu G, Salomonis N, Wang H, Williams A (2005). Anosva: a statistical method for detecting splice variation from expression data. Bioinformatics.

[24] Sandberg R, Neilson JR, Sarma A, Sharp PA, Burge CB (2008). Proliferating cells express mrnas with shortened 3’ utrs and fewer microrna target sites. Science.

[25] Laajala E, Aittokallio T, Lahesmaa R, Elo L (2009). Probe-level estimation improves the detection of differential splicing in affymetrix exon array studies. Genome Biol.

[26] Purdom E, Simpson KM, Robinson MD, Conboy JG, Lapuk AV, Speed TP (2008). Firma: a method for detection of alternative splicing from exon array data. Bioinformatics.

[27] Irizarry R, Hobbs B, Collin F, Beazer-Barclay K, Antonellis Y, Scherf U, Speed T (2003). Exploration, normalization, and summaries of high density oligonucleotide array probe level data. Biostatistics.

[28] Seok J, Xu W, Davis RW, Xiao W (2015). Rasa: Robust alternative splicing analysis for human transcriptome arrays. Sci Rep.

[29] Rodrigo-Domingo M, Waagepetersen R, Bodker JS, Falgreen S, Kjeldsen MK, Johnsen HE, Dybkaer K, Bogsted M (2013). Reproducible probe-level analysis of the affymetrix exon 1.0 st array with r/bioconductor. Brief Bioinform.

[30] Affymetrix. Genechip exon array design: Affymetrix Technical Note; 2005.

[31] Manhong D, Wang P, Boyd AD, Kostov G, Athey B, Jones EG, Bunney WE (2005). Evolving gene/transcript definitions significantly alter the interpretation of genechip data. Nucleic Acids Res.

[32] Talloen W, Clevert D, Hochreiter S, Amaratunga D, Bijnens L, Kass S, Göhlmann WHH (2007). I/NI Calls for the exclusion of non-informative genes: a highly effective filtering tool for microarray data. Bioinformatics.

[33] Kasim A, Lin D, Sanden SV, Clevert D, Bijnens L, Göhlmann H, Amaratunga D, Hochreiter S, Shkedy Z, Talloen W (2010). Informative or noninformative calls for gene expression: a latent variable approach. Stat Appl Genet Mol Biol.

[34] Bengtsson H, Irizarry R, Carvalho B, Speed T (2008). Estimation and assessment of raw copy numbers at the single locus level. Bioinformatics.

[35] Benjamini Y, Hochberg Y (1995). Controlling the false discovery rate: a practical and powerful approach to multiple testing. J R Stat Soc B Met.

[36] Shah SH, Pallas JA (2009). Identifying differential exon splicing using linear models and correlation coefficients. BMC Bioinformatics.

[37] Uhlén MEA (2015). Tissue-based map of the human proteome. Science.

[38] Kasim A, Lin D, Van Sanden S, Clevert D, Bijnens L, Goehlmann HWH, Amaratunga D, Hochreiter S, Shkedy Z, Talloen W (2010). Informative or noninformative calls for gene expression: a latent variable approach. Stat Appl Genet Mol Biol.

[39] Gerstein MB, Rozowsky J, Yan K, Waterston R (2014). Comparative analysis of the transcriptome across distant species. Nature.

[40] Braunschweig U, Barbose-Morais NL, Pan Q, Nachman EN, Alipanahi B, Gonatopoulos-Pournatzis T, Frey B, Irimia M, Blencowe BJ. Widespread intron retention in mammals functionally tunes transcriptomes. Genome Res. 2014;24(11). doi:10.1101/gr.177790.114. 10.1101/gr.177790.114 PMC4216919 25258385

[41] Consortium SI (2014). A comprehensive assessment of rna-seq accuracy, reproducibility and information content by the sequencing quality control consortium. Nat Biotechnol.

