# Supplementary material for: A random effects model for the identification of differential splicing (REIDS) using exon and HTA arrays
Source: BMC Bioinformatics. 2017 May 25;18:273. doi: 10.1186/s12859-017-1687-8 (PMC5445373; doi:10.1186/s12859-017-1687-8)
Supplement: Supplementary file 1 — Supplementary Material. Supplemental examples and figures (PDF 31000 kb) [file 12859_2017_1687_MOESM1_ESM.pdf]

REIDS: Random Effects for the Identification of Differential Splicing Using Exon and HTA Arrays

Marijke Van Moerbeke, Adetayo Kasim, Willem Talloen, Joke Reumers, Hinrich W. H. Göhlmann and Ziv Shkedy

1 Introduction

This appendix consists of additional clarifications and examples which is mentioned in the main manuscript. Section 2 gives an overview of four different scenarios which can arise in the detection of alternative splicing. We complement the examples given of for the HTA data in section 5.3 in the manuscript with examples form the tissue data. In Section 3 we present additional material for the tissue data while Section 4 concludes this supplementary appendix with extra examples for the HTA data.

2 Random Effects Model for the Identification of Differential Splicing

Supplementary Figure 1 illustrates the four scenarios which are described in Section 4.1 in the manuscript.

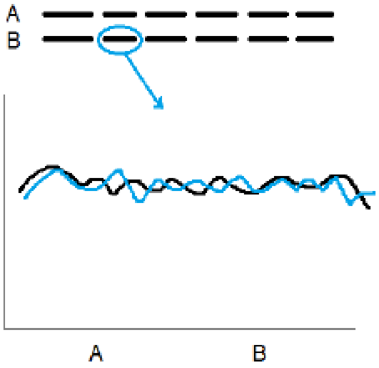

(a) A non-differentially expressed gene and no alternatively spliced exons. The gene and exon level of this exon k are similar across all arrays and no significant exon level deviation from the gene level is noticed. Therefore $\tau_k^2 < \sigma^2$. As a consequence, the exon score ρ_k will be low and the exon will not be identified as AS.

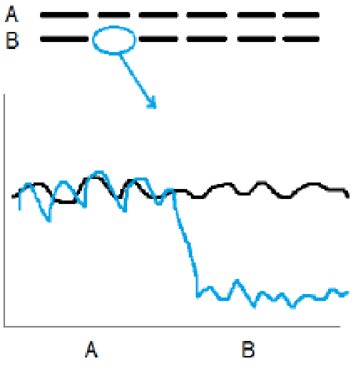

(b) A non-differentially expressed gene and an alternatively spliced exon. The gene level is similar across the arrays while the level of exon k differs between the groups. The exon is present in group A and depleted in group B which results in a deviation of the gene level there. Therefore, the variance across the arrays for this exon is expected to be higher than the variance of those exons that are not AS (indicated by $k-$): $\tau_k^2 > \tau_{k-}^2$ with $\tau_k^2 >> \sigma^2$ and $\tau_{k-}^2 << \sigma^2$. As a result the exon score ρ_k will be high. If the exon is included in group A their array scores will be around zero and if it is excluded in the group B, their array scores will be different than zero. A true difference will result in the identification of an alternatively spliced exon.

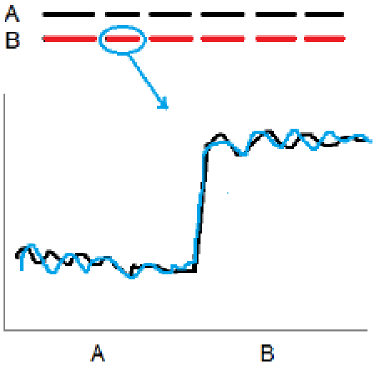

(c) A differentially expressed gene and no alternatively spliced exons. The gene and exon level are not similar across the arrays representing the differential expression of the gene. The exon level of exon k however does not deviate from the gene level. Since there is a natural difference between the gene levels of the arrays, it follows that $\tau^2 >> \sigma^2$ and that the exon score ρ_k is relatively high. A test on the array scores should however conclude that there is no alternatively splicing event as the deviations of the exon k from the gene level in both group A and goup B are relatively small.

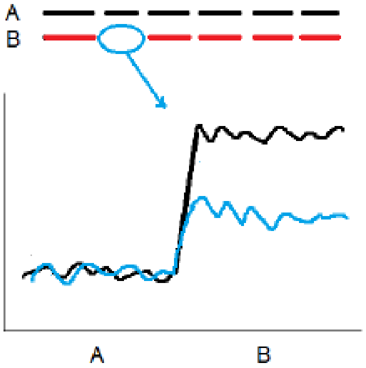

(d) A differentially expressed gene and an alternatively spliced exon. Both the gene and exon level of exon k are not similar across the arrays.In addition to the differential expression of the gene, the exon shows deviation of the gene level in group B as well. This implies that the exon is present in group A but depleted in group B. For all exons it is expected that $\tau_k^2 >> \sigma^2$ due to the natural difference and the deviation. A test of the array scores between group A and B should reveal those exons with a true deviation from the gene level in group B.

Supplementary Figure 1: *Illustration of the four scenarios which are considered in Section 4.1. Consider a gene consisting of several exons and two groups of samples A and B. Black lines refer to gene expression while blue lines refer to the expression level of an individual exon. Differentially expressed genes are indicated in red.*

The four scenarios are presented in Section 4.3 for the HTA data. Supplementary Figure 2 and 3 illustrate a differentially expressed gene with an alternatively spliced and a non-alternatively spliced exon respectively for the tissue data. We visualized probe set 2736397 in Supplementary Figure 2 which was mapped to a transcript cluster of the differentially expressed PDLIM5 gene. The probe set ranks at the third place in order of the exon scores with a score equal to 0.92 among the significant probe sets. Supplementary Figure 2 represents a gene that is differentially expressed considering the heart, muscle, thyroid and prostate tissues as group 1 and the remaining tissues as group 2. It is clear that probe set 2736397 is present in group 1 while it is depleted in group 2. The density plot of the array scores shows a bimodal distribution separating the groups of interest from each other.

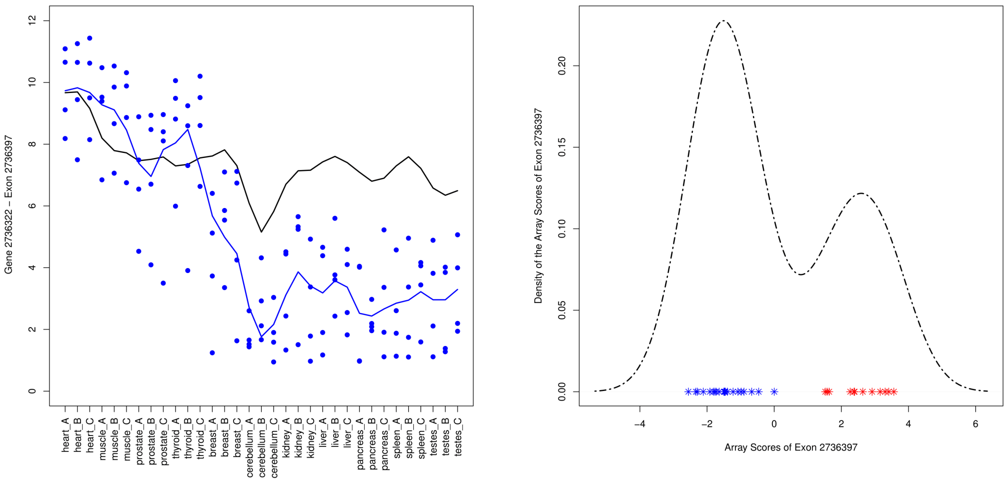

(a) The gene and exon expression levels.

(b) Density plot of the array scores.

Supplementary Figure 2: *Probe set 2736397. Left panel: gene level and exon level data. Black line: gene level data. Blue line: exon level data. Blue dots: probe level data. Right panel: a density plot for array scores showing the values of group 1 (red) and group 2 (blue).*

Supplementary Figure 3 shows probe set 3023189 of the non-differentially expressed gene FLNC which was selected for the tissue data. The probe set has an exon score of 0.12 which implies that the ratio between signal to the noise of the transcript cluster is relatively small. It is observed that the gene is differentially expressed between the groups of interest. Further, the exon levels lie close to their respective gene levels. This implies that these exons are present among all samples and that their deviation of the gene level is only natural. This is visualized in the density plots of the array scores where we expect an uni model distribution for a non-alternatively spliced probe set.

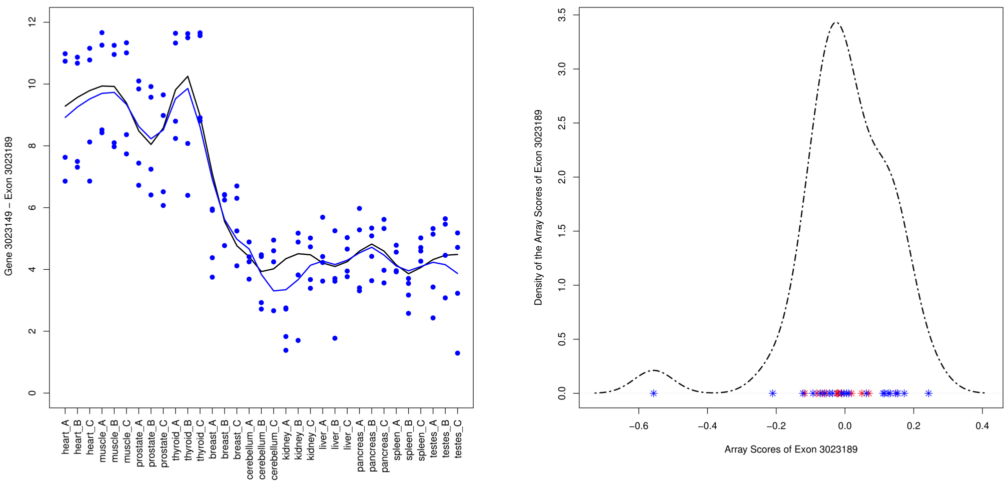

(a) The gene and exon expression levels. (b) Density plot of the array scores.

Supplementary Figure 3: *Probe set 3023189. Left panel: gene level and exon level data. Black line: gene level data. Blue line: exon level data. Blue dots: probe level data. Right panel: a density plot for array scores showing the values of group 1 (red) and group 2 (blue).*

Supplementary Figure 4 presents an example of a non-differentially expressed gene that has an alternatively spliced exon for the tissue data. We consider the groupings of the data as before. We selected probe set 3132962 with an exon score of 0.70. The probe set is significantly different between the groups of interest and is ranked at place 319. It has been mapped to belong to a transcript cluster of gene ANK1.

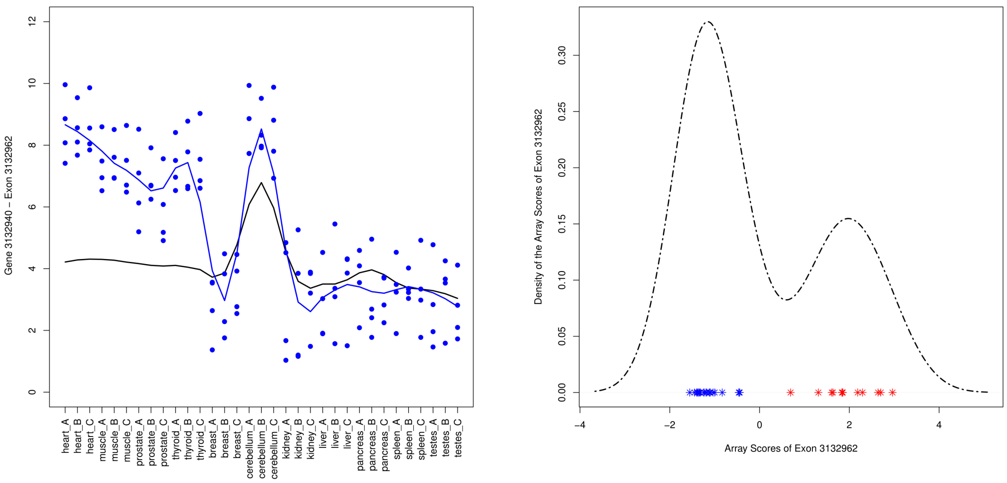

(a) The gene and exon expression levels. (b) Density plot of the array scores.

Supplementary Figure 4: *Probe set 3132962. Left panel: gene level and exon level data. Black line: gene level data. Blue line: exon level data. Blue dots: probe level data. Right panel: a density plot for array scores showing the values of group 1 (red) and group 2 (blue).*

It is observed that the gene ANK1. Probe set 3132962 however shows a large deviation of the gene level for the heart, muscle, thyroid and prostate tissue. This implies that this probe set has a higher inclusion in these tissues compared to the other tissues where the exon level matches with the gene level. When inspecting the density plots, we see a clear

bimodal distribution which represent a distinction between the groups of interest.

The last example we consider in this section, presented in Supplementary Figure 5 shows consists of a non-differentially expressed gene and a non-alternatively spliced exon. For the tissue data, probe set 4054185 belongs to a transcript cluster of the PPP1R2P3 gene. It has an exon score of 0.17 and it is not significantly different between the groups of the tissue data.

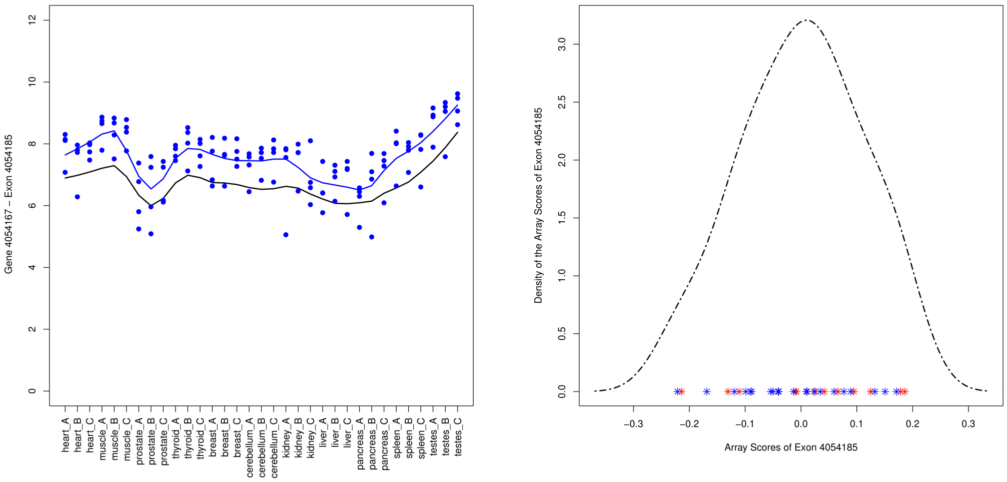

(a) The gene and exon expression levels.

(b) Density plot of the array scores.

Supplementary Figure 5: *Probe set 4054185. Left panel: gene level and exon level data. Black line: gene level data. Blue line: exon level data. Blue dots: probe level data. Right panel: a density plot for array scores showing the values of group 1 (red) and group 2 (blue).*

Further, the exon level values show only a little deviation of the gene level and do not indicate an alternative splicing event. The density plot of the array scores is a unimodal distribution and does not show a distinction between the groups of interest.

3 Tissue Data

Probe set 3307988 of the ABLIM1 gene is identified as alternatively spliced by the REIDS model and highlighted in Supplementary Figure 6. This figure shows all measured probe sets of the ABLIM1 gene and connects these to the known gene transcripts. The intensities of probe set 3307988 differ between the groups of interest of the tissue data. The declaration of this probe set as possibly alternatively spliced is supported by the visual illustration in 6.

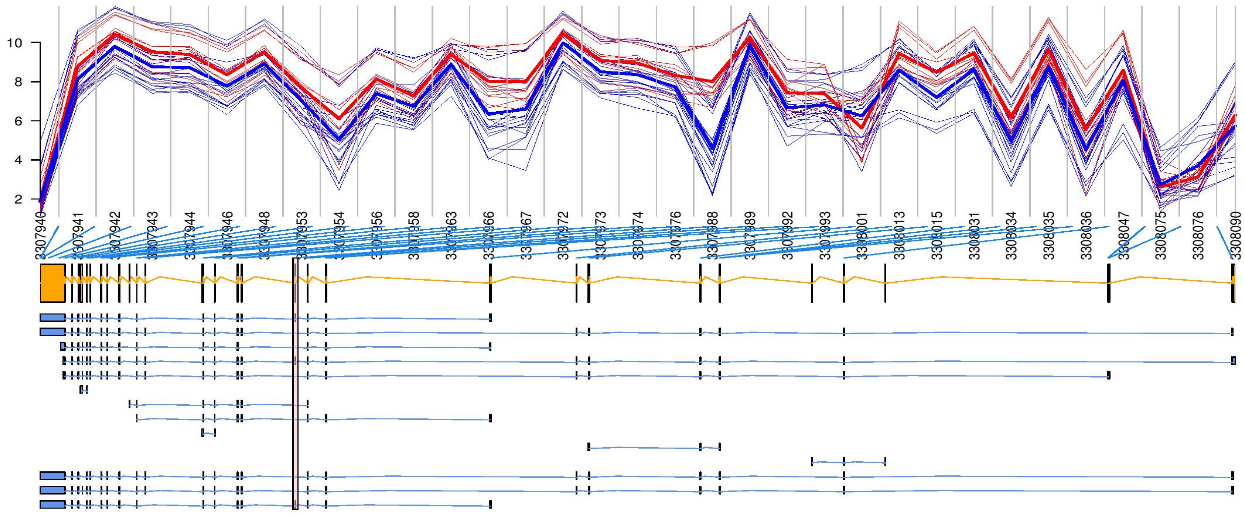

Supplementary Figure 6: *The measured intensities of the probe sets of the ABLIM1 gene with probe set 3307988 highlighted. The probe sets are annotated to the known transcripts of the ABLIM1 gene. The intensities of the heart, muscle, prostate and thyroid are shown in red while these of the other tissues are shown in blue.*

The top identified probe set 2513813 belongs to gene XIRP2 gene. Supplementary Figure 7 shows the gene and probe set expression over the different samples in the tissue data obtained with rma summarization. The probe intensities are as observed after preprocessing the data with background correction and normalization.

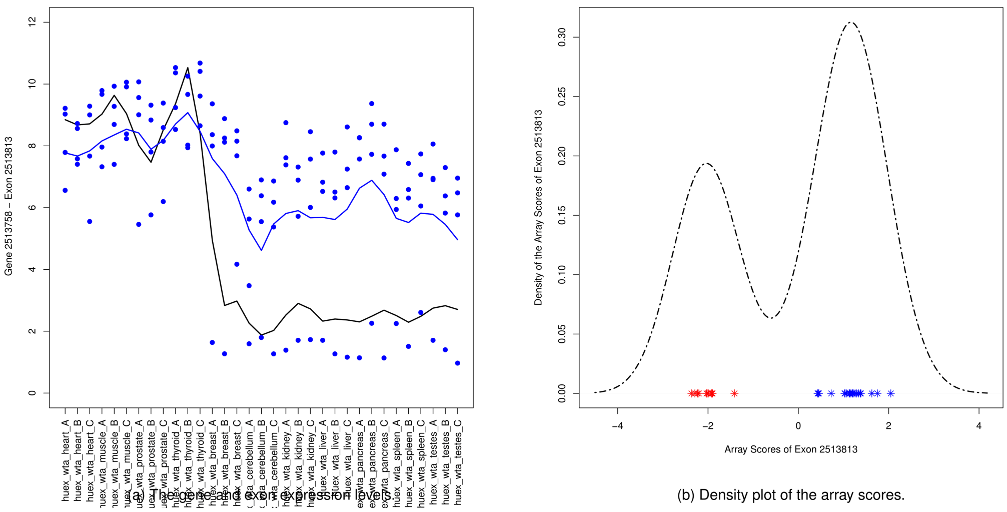

(a) The gene and exon expression levels

(b) Density plot of the array scores.

Supplementary Figure 7: *Probe set 2513813. Left panel: gene level and exon level data. Black line: gene level data. Blue line: exon level data. Blue dots: probe level data. Right panel: a density plot for array scores showing the values of group 1 (red) and group 2 (blue).*

The probe set does not show a large deviation from the gene level for these tissues. However, the probe set is showing an enrichment for the tissues of group 2. The density of the array scores obtained for probe set 2513813 are displayed in Supplementary Figure 7.

All probe sets and known transcripts of the XIRP2 gene are shown in Supplementary Figure **??** with a detailed plot in Supplementary Figure **??**. The candidacy of probe set 2513813 as alternatively spliced is supported by visual evidence. Furthermore, probe sets 2513807 and 2513823 are showing signs of differential splicing as well.

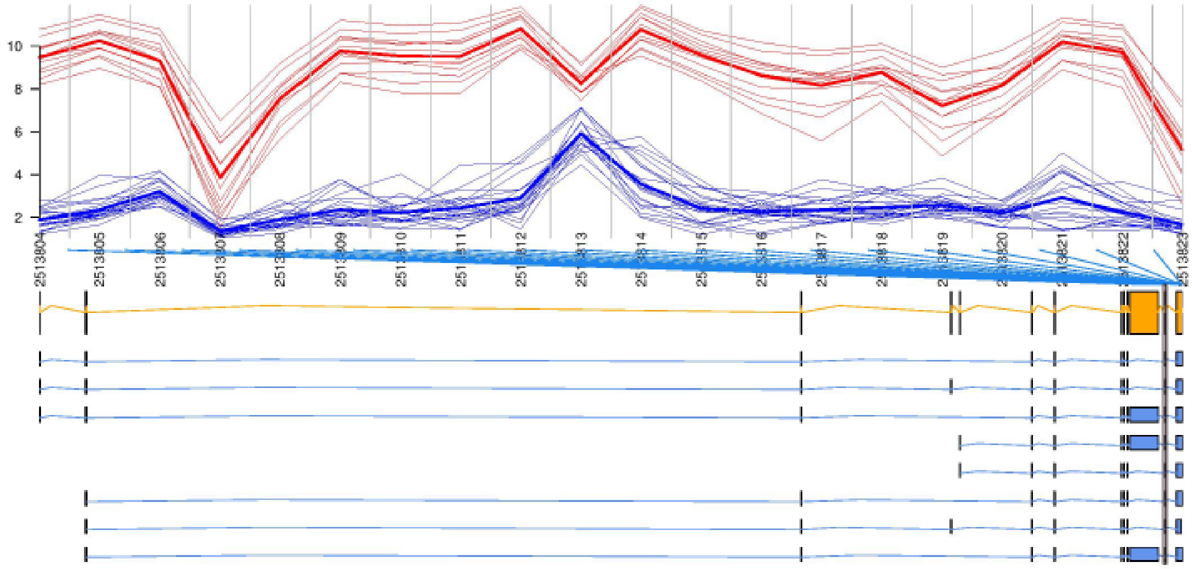

(a) The entire chromosomal region of the XIRP2 gene

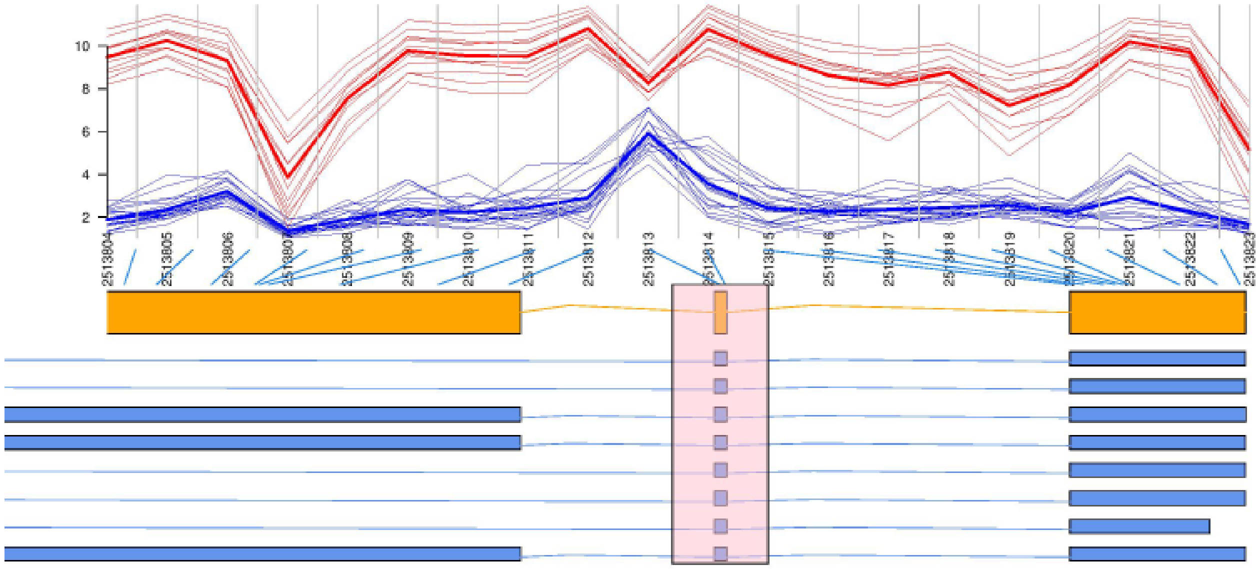

(b) A detailed region of the XIRP2 gene

Supplementary Figure 8: *The measured intensities of the probe sets of the XIRP2 gene with probe set 2513813 highlighted. The probe sets are annotated to the known transcripts of the XIRP2 gene. The intensities of the heart, muscle, prostate and thyroid are shown in red while these of the other tissues are shown in blue.*

A comparison between the array scores and FIRMA scores for XIRP2 is shown in Supplementary Figure 9. We observe a similarity in the patterns among the array scores and the FIRMA scores.

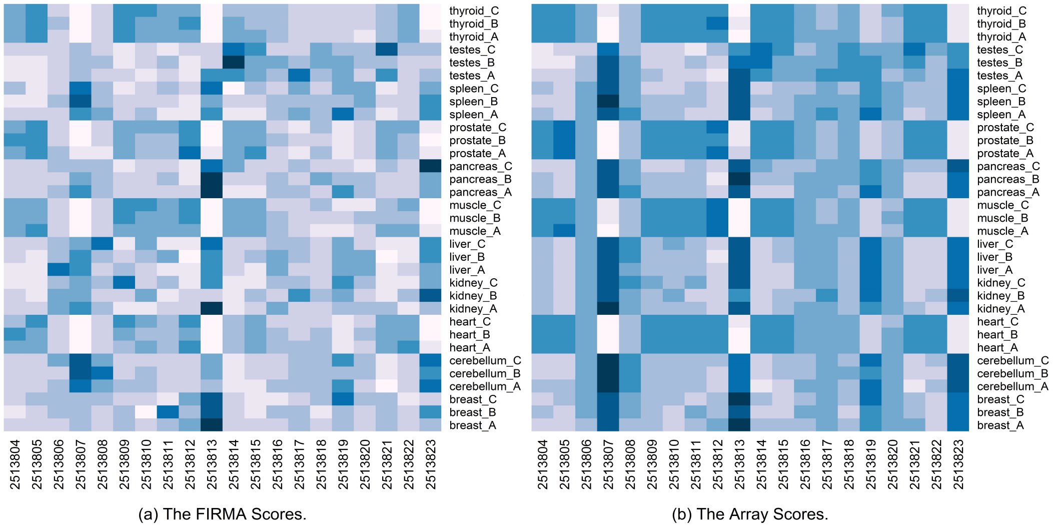

Supplementary Figure 9: *Left panel: a heatmap of the FIRMA scores of the XIRP2 gene. Right panel: a heatmap of the array scores of the XIRP2 gene.*

A search was conducted on genes known which have previously been reported to be differentially expressed in several cancer studies via the Expression Atlas website (`https://http://www.ebi.ac.uk/`). The search returns a number of experiments involving the queried gene. We investigated these genes in terms of alternative splicing by looking into their results of the *REMAS* model as presented in Table 1.

Table 1: *Differentially expressed genes for which alternative splicing is detected.*

Gene	Description
KIF1B	The KIF1B gene is found to be differentially expressed in 32 cancer studies covering several cancer types and tissues. The gene has been reported to be alternatively spliced in heart, muscle and thyroid (1). The probe sets with the highest exon scores are 2319718 and 2319121 with scores higher than 0.80 and are identified as alternatively spliced by the *REMAS* model.
PDE4DIP	A differential expression of the PDE4DIP gene has been reported in more than 70 cancer experiments. It contains 10 probe sets with exon scores higher than 0.7 but probe set 2432028 shows the most significant p-value when testing the array scores between the tissue groups.
TPM3	The TPM3 gene was observed to be differentially expressed in 18 cancer experiments. Five probe sets have been found to be the most significantly deviating from their respective gene levels between the tissue groups. These are the probe sets 2436538, 2436539,2436564, 2436565 and 2436566 with respecytively the exon scores 0.84, 0.79, 0.84, 0.87 and 0.87.
TNNI1	Four experiments have seen differential expression of the gene TNNI1. The probe set 2450832 passes the threshold for the exon score with a value of 0.60 and is identified as alternatively spliced.
MAP4	The MAP4 gene was discovered by 19 cancer studies. It has a probe set, 2673022, with an exon score higher than 0.9 and that shows a significant difference in the array scores between the groups of interest.
PDLIM5	Differential expression of the gene PDLIM5 was reported by 87 cancer studies. Probe set 2736397 has an exon sore of 0.92 and is identifief as alternatively spliced.
PALLD	The PALLD gene has been seen to be differentially expressed in 75 cancer experiments. Two probe sets, 2751072 and 2751068, have been identified as alternatively spliced with exon scores 0.69 and 0.67 respectively.
SYNPO2L	Four cancer experiments have reported gene SYNPO2L to be differentially expressed. Probe set 3294673 has an exon sore of 0.69 and is identified as alternatively spliced with the most significance.
SORBS1	The gene SORBS1 was shown by 51 experiments involving cancer to be differentially expressed. Three significant probe sets have been identified with exon scores higher than 0.80.
FHL1	The FHL1 gene was reported to be differentially expressed in 128 cancer related experiments. Probe set 3992432 with an exon score of 0.72 was identified to be alternatively spliced with the most significance.

Below we illustrate the genes KIF1B and PALLD of which probe sets 319718 and 2751068 respectively are identified as alternatively spliced by the REIDS model in the main manuscript. Both probe sets are supported by visual evidence as possible alternatively spliced.

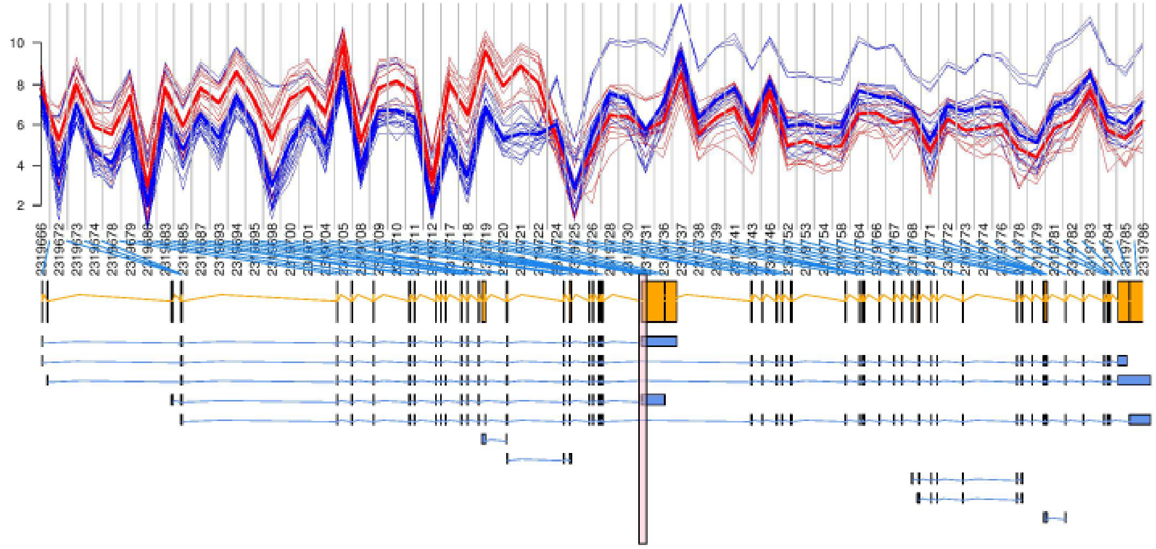

(a) The entire chromosomal region of the KIF1B gene

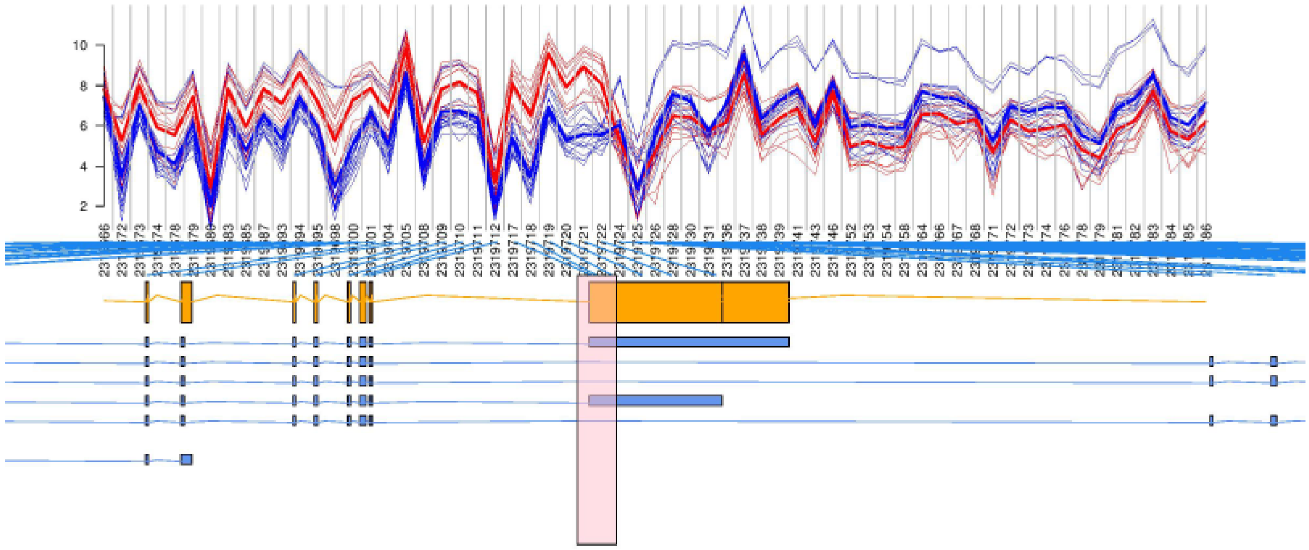

(b) A detailed region of the KIF1B gene

Supplementary Figure 10: *The measured intensities of the probe sets of the KIF1B gene with probe set 319718 highlighted. The probe sets are annotated to the known transcripts of the KIF1B gene. The intensities of the heart, muscle, prostate and thyroid are shown in red while these of the other tissues are shown in blue.*

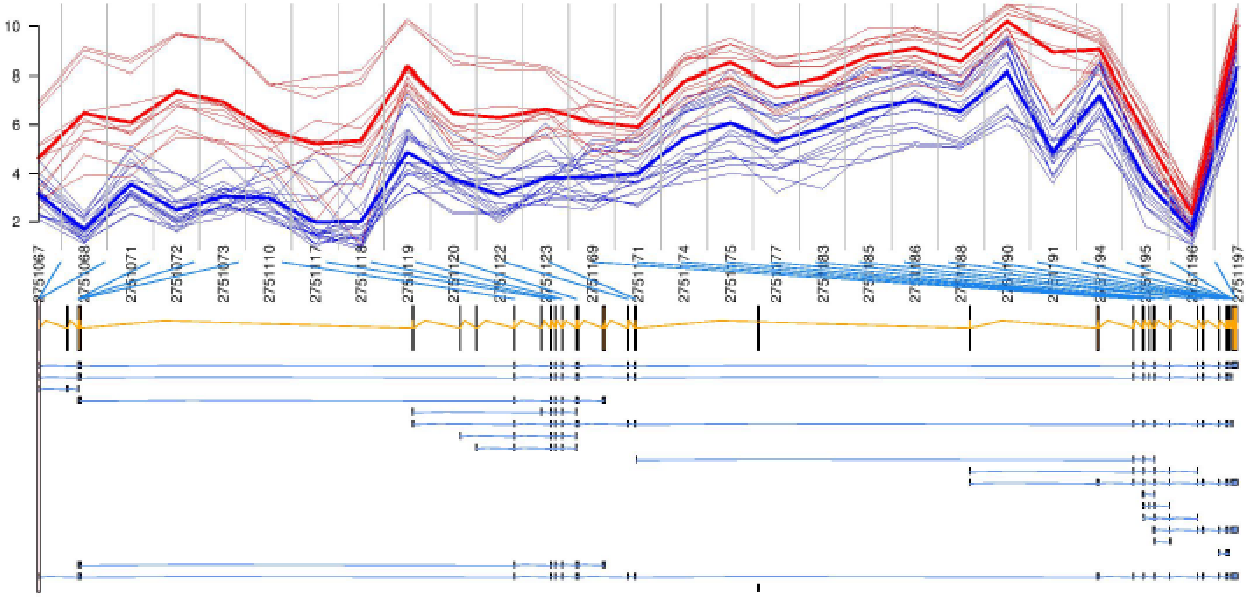

(a) The entire chromosomal region of the PALLD gene

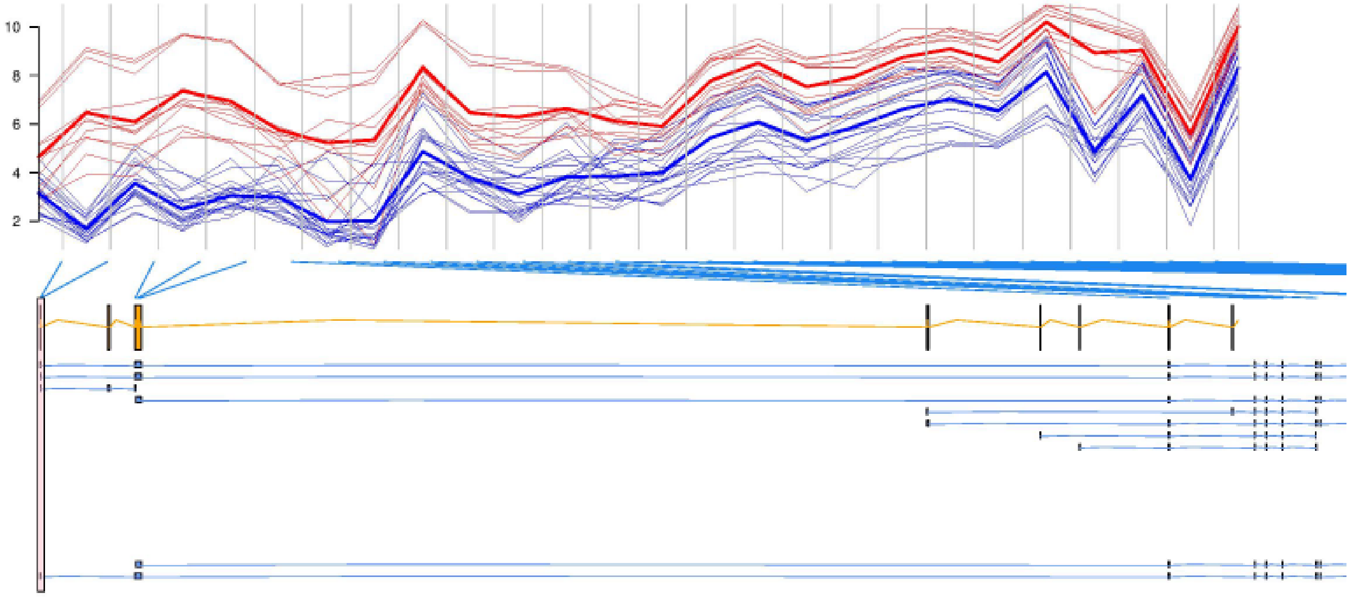

(b) A detailed region of the PALLD gene

Supplementary Figure 11: *The measured intensities of the probe sets of the PALLD gene with probe set 2751068 highlighted. The probe sets are annotated to the known transcripts of the PALLD gene. The intensities of the heart, muscle, prostate and thyroid are shown in red while these of the other tissues are shown in blue.*

4 The Colon Cancer Data

In this section we discuss the comparison between the FIRMA and the REIDS model in more detail. Supplementary Figure 12 shows the $-log10$(p-values) of FIRMA and REIDS.

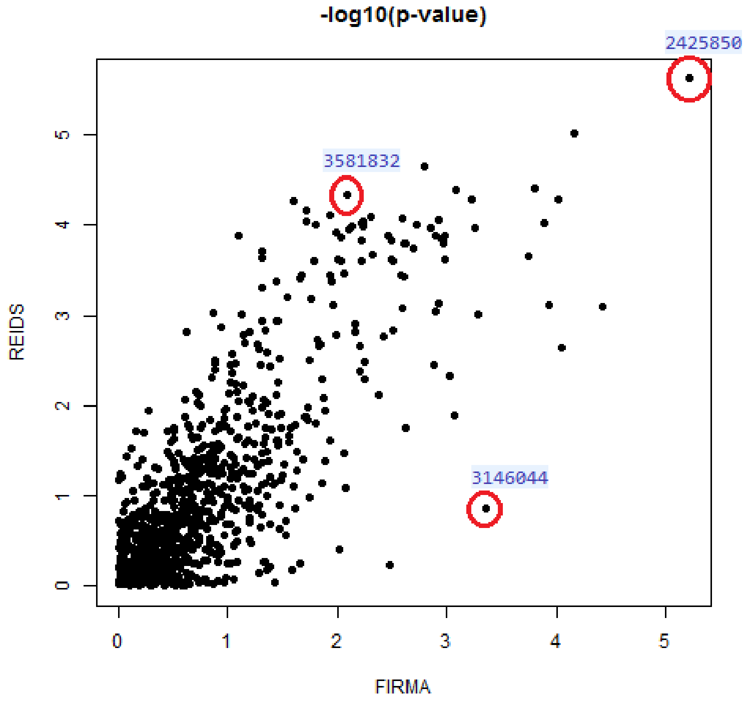

Supplementary Figure 12: *The $-log10(p-values)$ of FIRMA and REIDS.*

One case (the point in the upper right corner) is identified by both methods as alternatively spliced. This event is identified as exon 2425850 of the COL11A1 gene (Supplementary Figure 13 which has been observed to play an important role in the development of colon cancer [2].

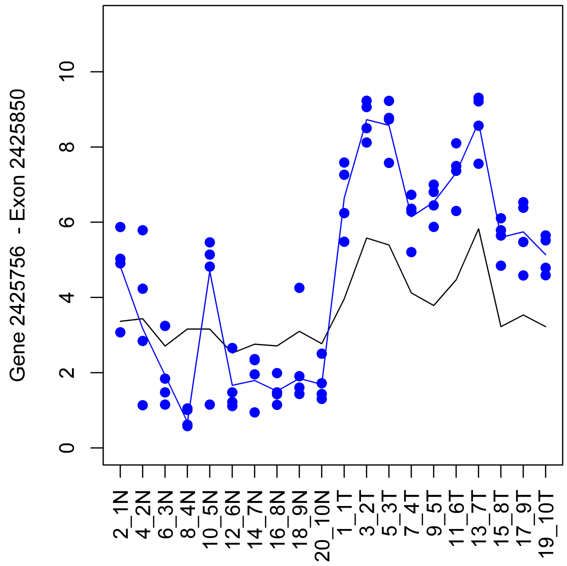

Supplementary Figure 13: *Probe set 2425850 of the COL11A1 gene. The black line and blue lines indicate the mean profiles of the gene and exon level data respectively. The blue dots show the probe level data.*

We now focus on exon 3581832 of the ELK2A gene. This exon has a high rank for the REIDS p-values and a lower rank for the FIRMA p-values. Supplementary Figure 15 shows that the exon level expression is enriched for the tumor samples.

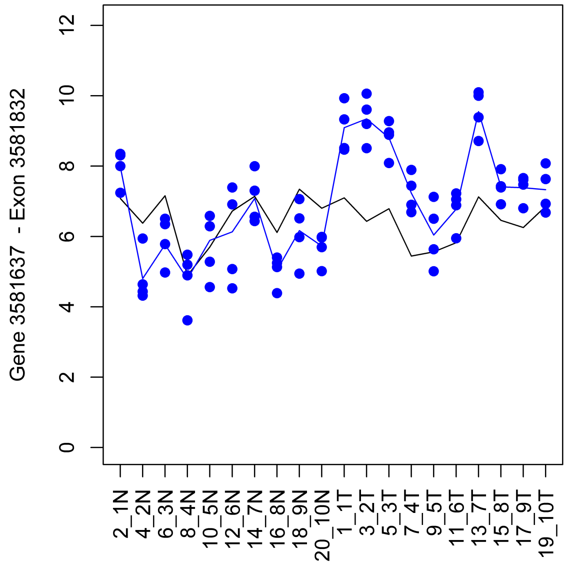

Supplementary Figure 14: *Probe set 3581832 of the ELK2A gene. The black line and blue lines indicate the mean profiles of the gene and exon level data respectively. The blue dots show the probe level data.*

Exon 3146044 of the NIPAL2 gene has a high rank in the FIRMA p-values but substantially lower in the REIDS p-values. The exon is represented by only 1 probe and therefore the measured value might be inaccurate.

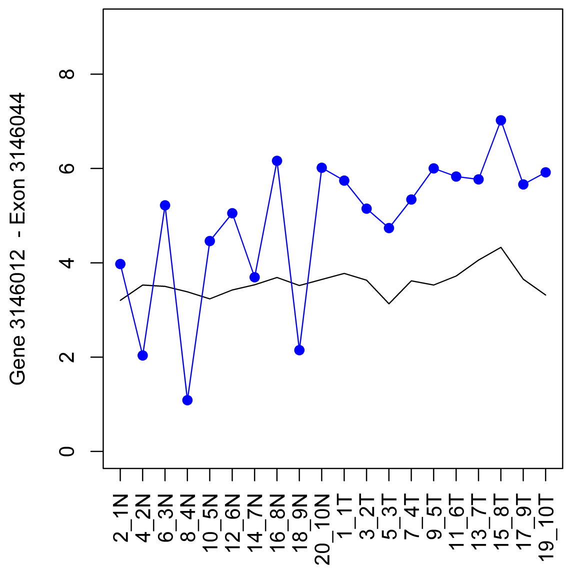

Supplementary Figure 15: *Probe set 3146044 of the NIPAL2 gene. The black line and blue lines indicate the mean profiles of the gene and exon level data respectively. The blue dots show the probe level data.*

We note that the FIRMA method does not provide p-values when the method is performed. Rather, the scale of the residual is investigated to identify those that are outlying. We have introduced a t-test on the FIRMA scores to be able to identify alternatively spliced exons by means of one measure.

5 HTA Data

The spectral map motivating the choice of the groups for the HTA data is presented in Supplementary Figure 16.

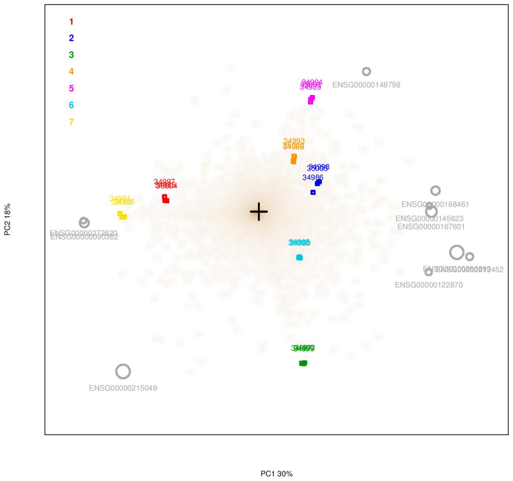

Supplementary Figure 16: *Spectral map on the gene level of the HTA data.*

Group 1 and group 7 represent the colon cancer samples. Group 2 and 5 are the lung cancer cell lines. Groups 3, 4 and 6 are, respectively, the ovary, prostate and breast cancer cell lines. The top probe set is ENSE00001668645 and has an exon score of 0.70. The corresponding transcript cluster belongs to the DOCK10 gene which has been reported in several cancer studies, not limited to only colon cancer. Supplementary Figure 17 shows the gene and exon expression summarized with the rma procedure for the samples of the HTA data. The plot shows an enrichment of the probes for the second group of cell lines.

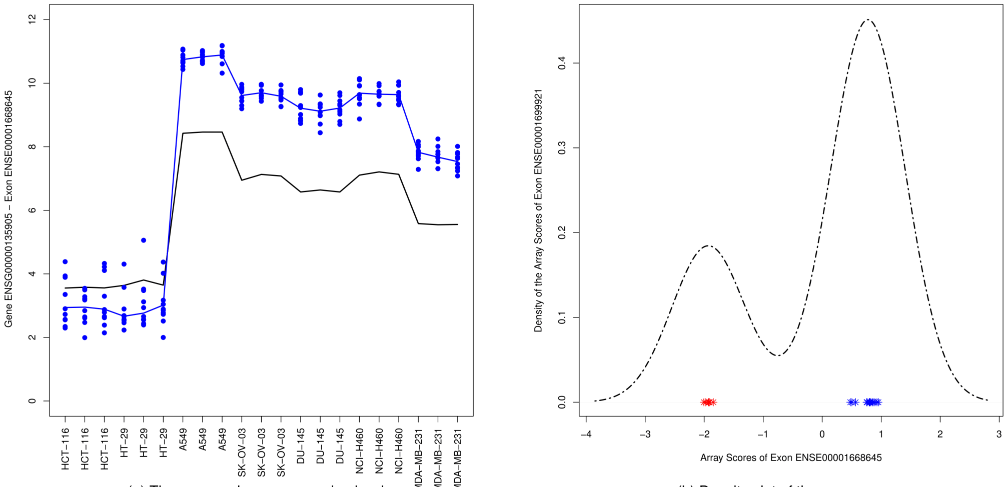

(a) The gene and exon expression levels.

(b) Density plot of the array scores.

Supplementary Figure 17: *Probe set ENSE00001668645. Left panel: gene level and exon level data. Black line: gene level data. Blue line: exon level data. Blue dots: probe level data. Right panel: a density plot for array scores showing the values of group 1 (red) and group 2 (blue).*

The density plot of the array scores in Supplementary Figure 17 has a bimodal tendency and shows a clear separtion between the colon cancer samples and the other cell lines. All measured probe sets of the DOCK10 gene and their annotation to known gene transcripts is displayed in Supplementary Figure **??**. A detailed region is shown in Supplementary

Figure ??. Probe set ENSE00001668645 is highlighted and while the intesities for the colon cancer cell line decrease, the intensities of the ther cell lines show a higher increase.

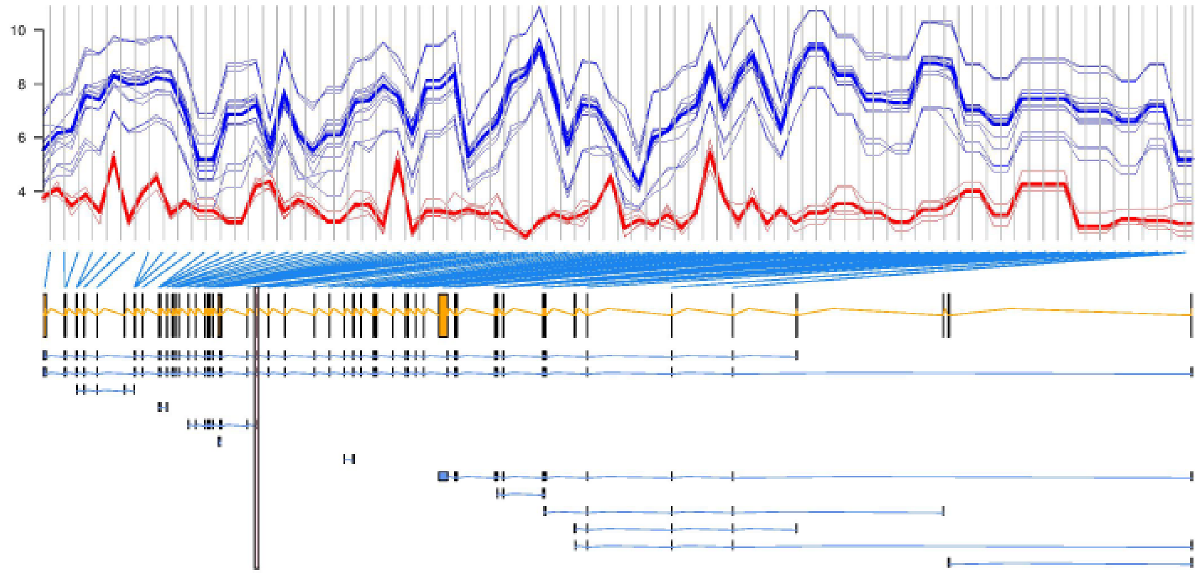

(a) The entire chromosomal region of the DOCK10 gene

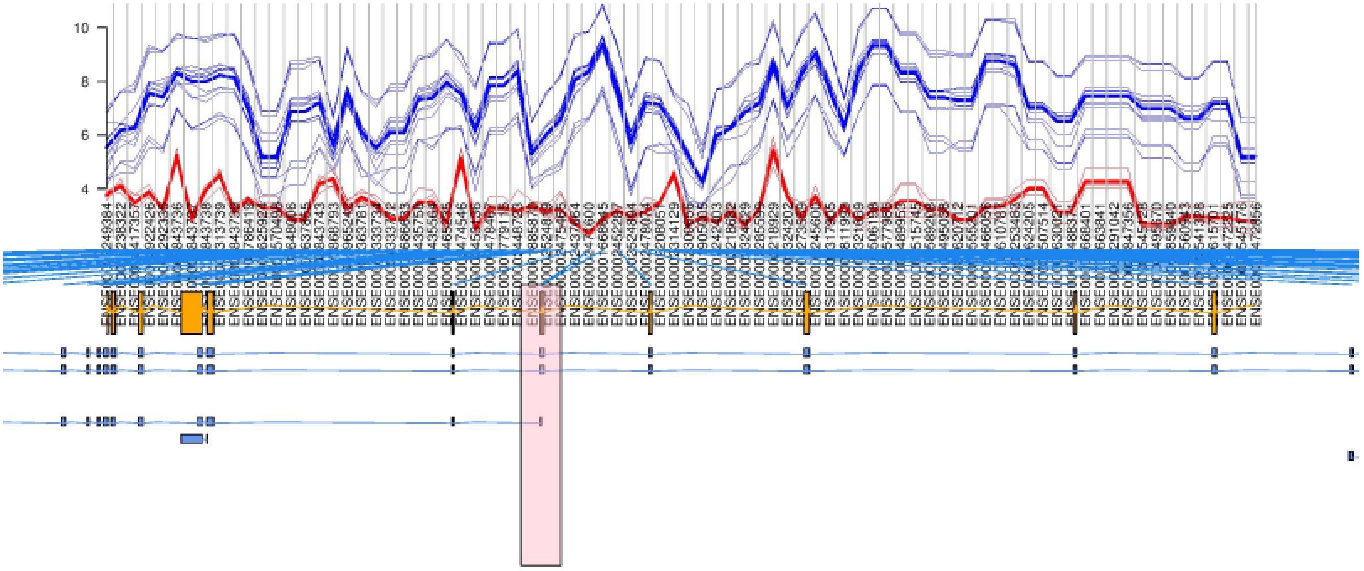

(b) A detailed region of the DOCK10 gene

Supplementary Figure 18: *The measured intensities of the probe sets of the DOCK10 gene with probe set ENSE00001668645 highlighted. The probe sets are annotated to the known transcripts of the DOCK10 gene. The intensities of the colon cancer cell lines are shown in red while these of the other cell lines are shown in blue.*

A heatmap of the array and FIRMA scores is presented in Supplementary Figure 19. We observe similar patterns among the array scores and the FIRMA scores for the probe set ENSE00001668645, as before FIRMA scores seems to have a higher variability than the *REMAS* array scores.

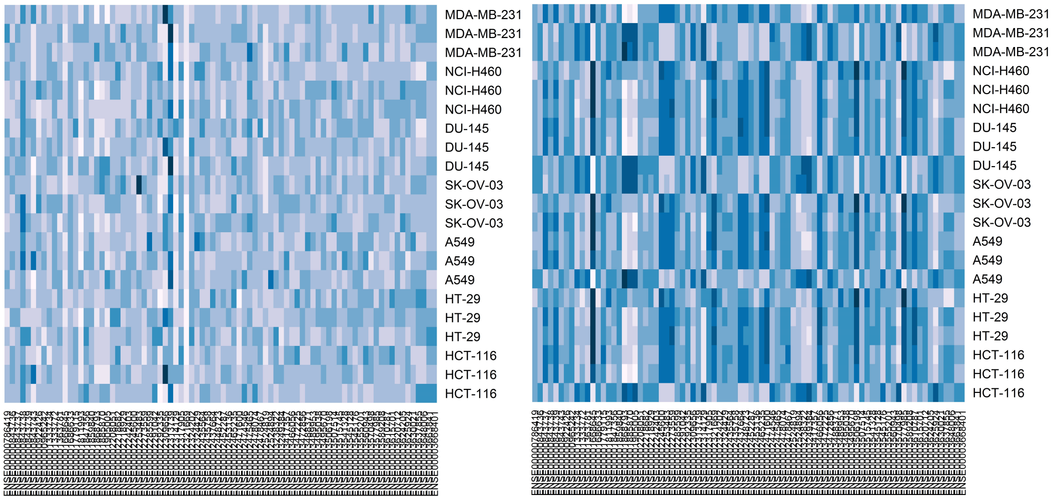

(a) FIRMA Scores. (b) Array Scores.

Supplementary Figure 19: *Left panel: a heatmap of the FIRMA scores of the DOCK10 gene. Right panel: a heatmap of the array scores of the DOCK10 gene.*

We illustrate the annotation of the probe sets of the MYO18A gene which was mentioned in the main manuscript as an example of a DE gene with an AS exon. Supplementary Figure 20 illustrates the alternative splicing of the probe set ENSE00001297204.

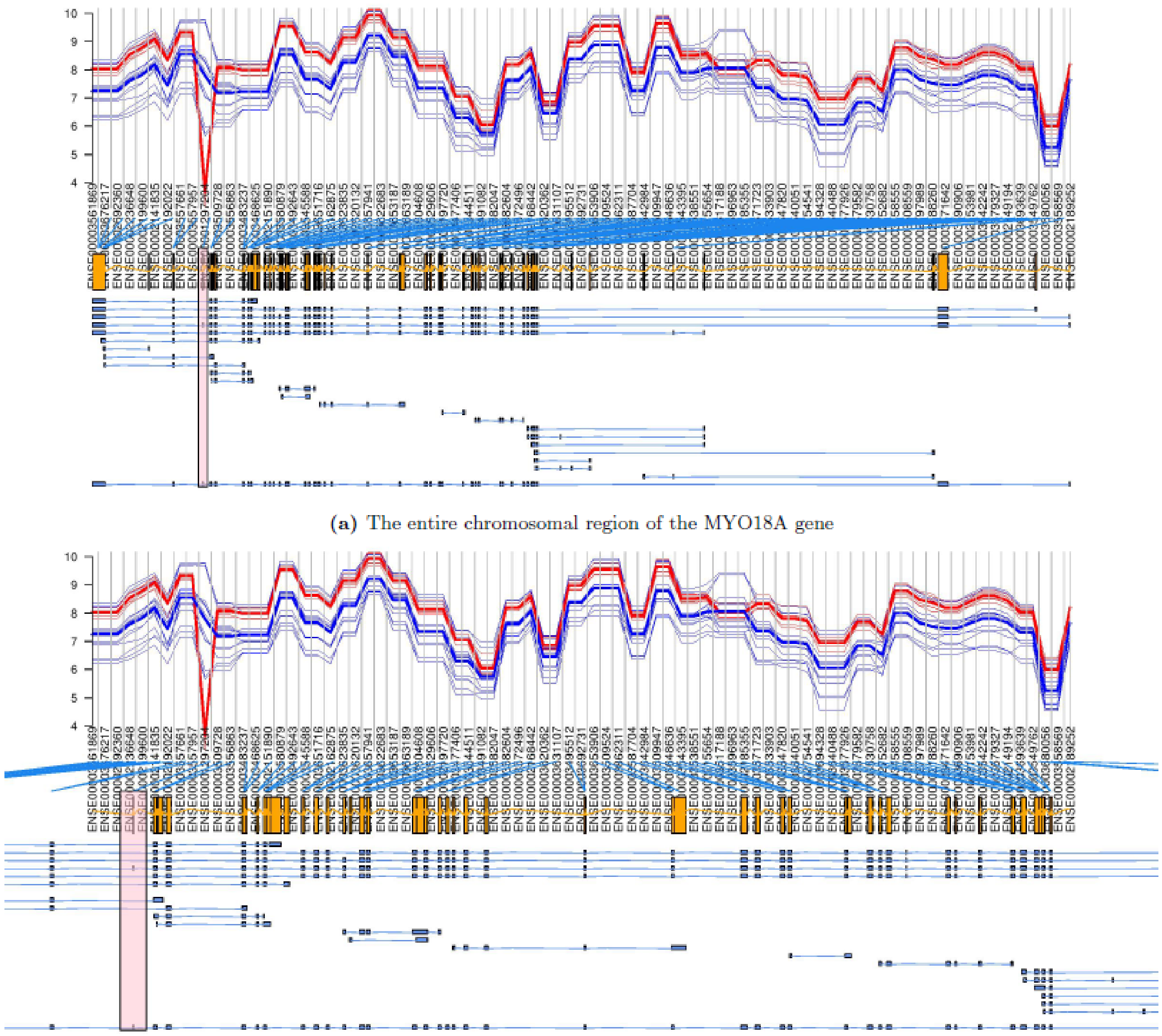

(a) The entire chromosomal region of the MYO18A gene

(b) A detailed region of the MYO18A gene

Supplementary Figure 20: *The measured intensities of the probe sets of the MYO18A gene with probe set ENSE00001297204 highlighted. The probe sets are annotated to the known transcripts of the MYO18A gene. The intensities of the colon cancer cell lines are shown in red while these of the other cell lines are shown in blue.*

References

[1] Shah S H, Pallas J A. Identifying differential exon splicing using linear models and correlation coefficients. BMC Bioinformatics. 2009;10(26).

[2] Fischer H, Stenling R, Rubio C, Lindblom A. Colorectal carcinogenesis is associated with stromal expression of COL11A1 and COL5A2. Carcinogenesis. 2001;22(6):875–878.
